# Development and validation of asthma risk prediction models using co-expression gene modules and machine learning methods

**DOI:** 10.1038/s41598-023-35866-2

**Published:** 2023-07-12

**Authors:** Eskezeia Y. Dessie, Yadu Gautam, Lili Ding, Mekibib Altaye, Joseph Beyene, Tesfaye B. Mersha

**Affiliations:** 1 Department of Pediatrics, Cincinnati Children’s Hospital Medical Center, University of Cincinnati College of Medicine, Cincinnati, OH USA; 2 Department of Health Research Methods, Evidence, and Impact, McMaster University, Hamilton, Canada

**Keywords:** Computational models, Gene expression

## Abstract

Asthma is a heterogeneous respiratory disease characterized by airway inflammation and obstruction. Despite recent advances, the genetic regulation of asthma pathogenesis is still largely unknown. Gene expression profiling techniques are well suited to study complex diseases including asthma. In this study, differentially expressed genes (DEGs) followed by weighted gene co-expression network analysis (WGCNA) and machine learning techniques using dataset generated from airway epithelial cells (AECs) and nasal epithelial cells (NECs) were used to identify candidate genes and pathways and to develop asthma classification and predictive models. The models were validated using bronchial epithelial cells (BECs), airway smooth muscle (ASM) and whole blood (WB) datasets. DEG and WGCNA followed by least absolute shrinkage and selection operator (LASSO) method identified 30 and 34 gene signatures and these gene signatures with support vector machine (SVM) discriminated asthmatic subjects from controls in AECs (Area under the curve: AUC = 1) and NECs (AUC = 1), respectively. We further validated AECs derived gene-signature in BECs (AUC = 0.72), ASM (AUC = 0.74) and WB (AUC = 0.66). Similarly, NECs derived gene-signature were validated in BECs (AUC = 0.75), ASM (AUC = 0.82) and WB (AUC = 0.69). Both AECs and NECs based gene-signatures showed a strong diagnostic performance with high sensitivity and specificity. Functional annotation of gene-signatures from AECs and NECs were enriched in pathways associated with IL-13, PI3K/AKT and apoptosis signaling. Several asthma related genes were prioritized including SERPINB2 and CTSC genes, which showed functional relevance in multiple tissue/cell types and related to asthma pathogenesis. Taken together, epithelium gene signature-based model could serve as robust surrogate model for hard-to-get tissues including BECs to improve the molecular etiology of asthma.

## Introduction

Asthma is a complex heterogeneous disease characterized by recurring symptoms of reversible airflow obstruction, bronchial hyperresponsiveness, and airway inflammation. Genetic, environmental and other social determinants risk factors play key roles in asthma etiology^1^. A family history of asthma is an associated with an increase in asthma risk in the offspring, demonstrating a strong genetic component with estimated heritability as high as 80%^2,3^. Clinical outcome such as lung function, Immunoglobulin and skin prick test have been suggested as clinical biomarker. However, clinical biomarkers are not specific to capture the allergic inflammation signal presented in asthmatic patients. There is a need for an effective molecular biomarker that are closely linked to disease mechanisms. Gene expression profiling techniques that simultaneously analyze a large quantity of transcripts are well suited to identify novel genes and pathways involved in asthma pathogenesis^4,5^. Transcriptomic signatures that differentiate asthmatic and healthy state based on genome-wide gene expression profile in different human tissue/cell types including nasal airway epithelium cells (NECs), airway epithelium cells (AECs), bronchial airway epithelium cells (BECs), and whole blood (WB) cells were reported previously^6^. Furthermore, gene signatures from airway smooth muscle (ASM) cells data that discriminate asthmatic and healthy subjects were reported^7^. Ideally, gene expression based diagnostic model should be constructed in the tissue/cell types relevant to the disease development^8^. For example, identifying biomarkers and constructing diagnostic genetic models using bronchial airway epithelium as target tissue have great potential for elucidating pathophysiological changes in bronchial airways of asthma^8^. However, one of the major challenges in asthma research is obtaining sufficient bronchial epithelial samples to construct diagnostic and prediction models. This is not realistic specifically in children because obtaining these samples requires performance of invasive bronchoscopies.

Previous studies demonstrated that nasal upper bronchial airway epithelium tissue/cell types shared the same airway biology with lower respiratory tracts^8–10^. Thavagnanam et al. suggested to use easily accessible tissue (e.g., NECs) as a surrogate for less accessible tissue (e.g., bronchial epithelium) in asthma studies^6^. The WB cells is another surrogate sample and used in many asthma studies. In addition, samples obtained from easily accessible surrogate tissue can help to get large sample size required for developing diagnostic model with sufficient statistical power. Identifying gene signatures obtained from easily accessible tissue sampled during asthma attacks can aid to elucidate the pathogenesis and changes in asthmatic easy-to-access bronchial airways^11,12^. However, previous studies have typically considered a single tissue or a small number of tissues and there are limited studies that comprehensively evaluated whether diagnostic models derived from surrogate tissue samples can provide comparable diagnostic performance with less accessible target tissues (i.e., cells from lung tissue). In this study, we systematically determine which of the genes have tissue-specific effects or broadly shared among tissue types.

In addition, most of the previous studies in asthma mainly focused on identifying DEGs. The analysis and interpretation of DEGs is important for defining key genes which may be driving changes in asthma. However, individual genes may fail to fully capture the molecular pathways as genes do not function in isolation. Most importantly, each gene has limited contribution to complex diseases including asthma^13^. Co-expression analysis considers all genes together and constructs networks among genes to form co-expressed modules and these modules are potentially used to infer regulatory association between target genes and transcription factors^14^. Furthermore, co-expression module genes can be used to discover interaction network and hidden biological models relevant to disease pathogenesis^15,16^.

In recent years, machine learning approaches such as LASSO logistic regression, SVM and random forest (RF) were used to unravel new biological insights from the genomic data^17,18^. Although these approaches identified potential gene signatures in asthma, their approach only considered each gene individually. However, the individual genes may not always decipher meaningful biological functions. To address these limitations, we analyzed gene expression data from 257 asthmatic and 136 control subjects in NECs and 62 asthmatic and 43 control subjects in AECs and developed co-expression network graph combined with machine learning methods to prioritize and select potential genes related to asthma risk. We further validated the performance of the risk models in an independent and different tissue types including BECs, ASM and WB datasets.

## Materials and methods

### Data sets and filtering criteria

**Figure 1:**
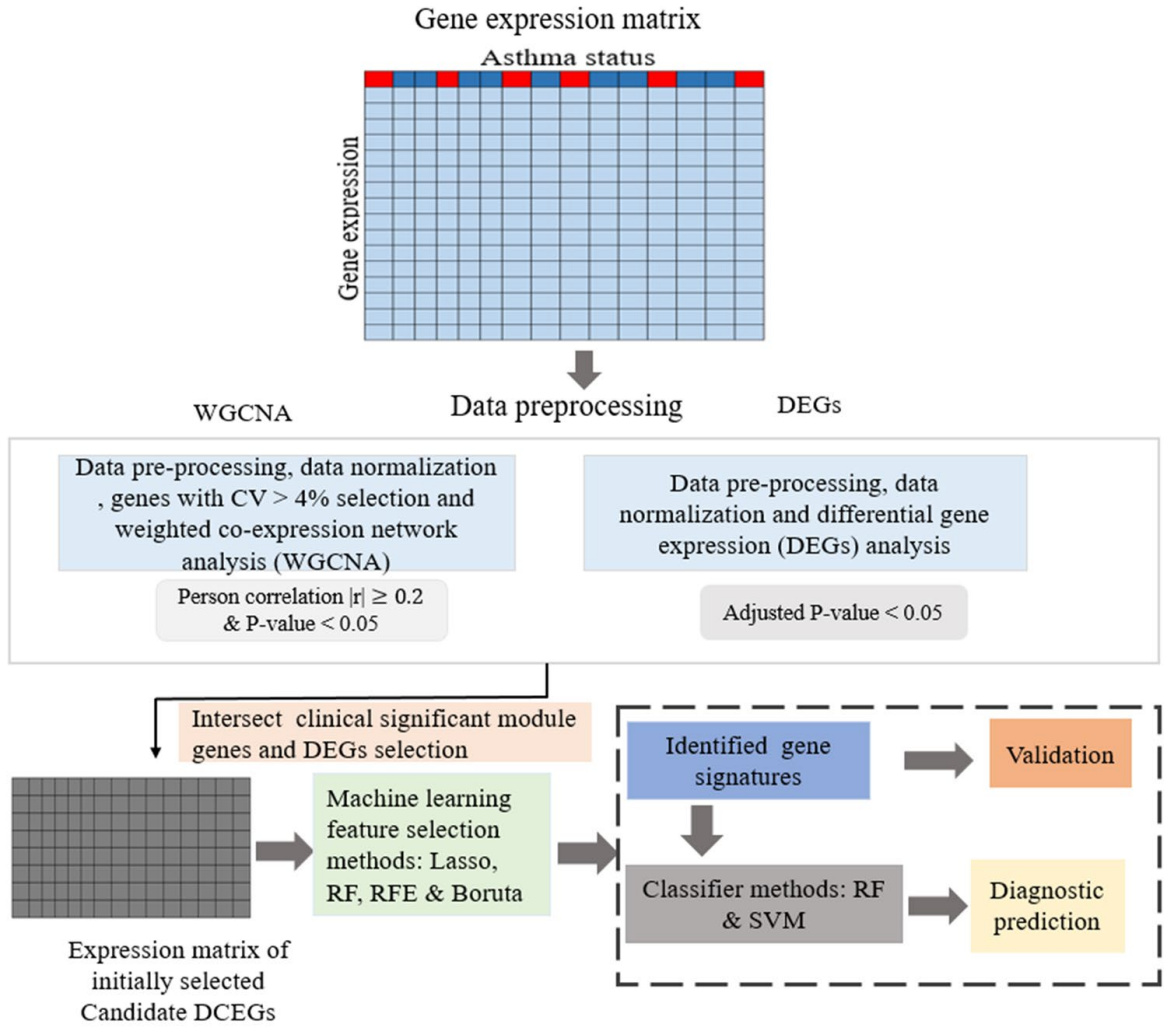
The overall workflow of this study. Initially, RNA-sequencing or microarray based gene expression data were collected, preprocessed, normalized, and analyzed for differential expression analysis and weighted correlation network analysis (WGCNA) to generate DEGs and modules associated with asthma status. To identify differentially co-expressed genes (DCEGs), an intersecting analysis between DEGs (adjusted *p*-value < 0.05) and genes within modules significantly correlated with asthma status (*P*-value < 0.05) was performed. Candidate DCEGs were further analyzed by four machine learning algorithms to identify gene-signatures and constructed different asthma classification models and prediction, which were then validated in independent datasets. CV-coefficient of variation.

Our overall workflow strategy is shown Fig. 1. Initially, gene expression profiles and the corresponding clinical information were downloaded from publicly available NCBI Gene Expression Omnibus (GEO) database. Eligible gene expression asthma datasets were chosen using the following inclusion criteria. (1) Homo sapiens, (2) sample size ≥ 50, (3) consists of gene expression profiles of asthmatic and control subjects and published within seven years. Three asthma RNA-seq datasets (accession: GSE152004, GSE201955 and GSE58434) and two asthma microarray datasets (accession: GSE67472 and GSE69683) satisfied the inclusion criteria were used for subsequent analysis. The raw count dataset, GSE152004 derived from nasal epithelium cells (NECs) contains 257 asthmatic and 136 control subjects. The normalized microarray dataset, GSE67472 derived from airway epithelium cells (AECs) contains 62 asthmatic and 43 control subjects. The normalized RNA-seq data in GSE201955 derived from bronchial epithelial cells (BECs) contains 79 asthmatic and 39 control subjects. The RNA-seq data in GSE58434 derived from airway smooth muscle (ASM) cells contains 17 asthmatic and 36 control subjects. The normalized microarray data in GSE69683 derived from whole blood (WB) cells contains 324 asthmatic and 87 control subjects. The summary of the datasets used in this study are shown Table 1.

**Table 1:** Asthma gene expression datasets used in current study.

Tissue class	GEO ID	Sample size (cases /controls)	Gender (% female)	Age (years), mean ± SD	Tissue type	Platform
Surrogate	GSE152004	257/136	51	14.35 $\pm$ 3.19	NECs	Illumina HiSeq 2000
Primary/target tissue	GSE67472	62/43	51	35.34 $\pm$ 10.83	AECs	Affymetrix Human Genome U133 Plus 2.0 Array
Surrogate	GSE69683	324/87	55	NA	WB	Affymetrix HT HG-U133 + PM Array
Primary	GSE201955	79/39	71	38.54 $\pm$ 12.07	BECs	Illumina HiSeq 2500 and 400
Primary	GSE58434	17/36	NA	NA	ASM	Illumina HiSeq 2000

*AECs* Airway epithelial cells, *ASM* Airway smooth muscle, *BECs* Bronchial epithelial cells, *NECs* Nasal epithelial cells, *WB* Whole blood.

### Data processing and selection of DEGs

For raw count RNA-seq expression matrix in the NECs (GSE152004) dataset, DESeq2^19^ package was used to pre-process, filter out genes showing less than 10 reads based on the sum of rows and normalize the background. Batch effect, treatment effect and/or unrelated variables in the datasets derived from ASMs and BECs were eliminated using surrogate variable analysis (SVA) package^20^. After each dataset was preprocessed and normalized separately, the normalized gene expression datasets derived from AECs and NECs tissue types were used for model development and the normalized datasets derived from BECs, ASM and WB tissue/cell type samples were used for model validation. Differentially expressed genes (DEGs) in asthmatic subjects compared with controls were identified using limma^21^ package and a significance threshold adjusted *P*-value < 0.05 based on Benjamin-Hochberg procedure was used to identify DEGs in AECs and NECs datasets. We used the ggplot2 package to generate a volcano plot and show both the adjusted *P*-value and fold change. The statistical software R was used to conduct all statistical analyses.

### Gene co-expression network analysis

Initially, genes were filtered by coefficient of variation (CV) to avoid non-varying or low-expressed genes in both AECs and NECs datasets, and genes with CV > 4% (5833 and 7496 hypervariable genes in AECs and NECs, respectively) were utilized to construct a gene coexpression network using WGCNA R package^22^. To characterize the correlation structure of these hypervariable genes, gene similarity matrix was constructed using pairwise correlation ${S}_{ij}$=$cor ({x}_{i}, {x}_{j}$), where ${x}_{i}$ and ${x}_{j}$ represent the ith row and the jth row of gene expression data matrix X, respectively. The similarity matrix was transformed into an adjacency matrix, represented by ${A}_{ij}= {\left|cor ({x}_{i}, {x}_{j})\right|}^{\beta }$, where the suitable soft-thresholding candidate power β that ranges from 1 to 20 and the appropriate power were determined based on index value in the dataset ( usually greater than 0.85) using the pick Soft Threshold function^22^. Second, the adjacency gene network was transformed into a topological overlap matrix (TOM), and corresponding dissimilarity (1-TOM) matrix was computed. Finally, average linkage hierarchical clustering with Dynamic Tree Cut was used to identify modules, and minimum number of genes in each module was set to be 50^23^.

### Selection of asthma correlated modules and differentially co-expressed genes (DCEGs)

To select asthma correlated modules, module eigengenes (ME), which is the principal component of each gene module and could be considered as a representative of all genes in a given module was computed. The ME values were correlated with asthmatic and control subjects using Pearson’s correlation and the modules significantly correlated with asthmatic subjects were selected (|r| $\ge$ 0.2 and *P*-value < 0.05). The genes within the modules that had significant association with asthmatic subjects and controls in AECs and NECs dataset were selected and named as module genes (co-expressed genes). Then, an overlapping analysis was conducted between co-expressed genes and DEGs to screen differentially co-expressed genes (DCEGs) for further analysis.

### Gene prioritization using four machine learning algorithms

For identification of prioritized gene-signatures associated with asthma, we used gene expression data from AECs (n = 105) and NECs (n = 393) and selected the respective DCEGs as input features in four different supervised ML algorithms: RF, recursive feature elimination (RFE), LASSO, and Boruta^24–27^.

RF is a supervised ML algorithm, which creates decision trees on randomly selected data samples, obtains prediction from individual tree and choose best solution by means of majority voting. RF also uses mean decrease accuracy for ranking individual gene-importance^24^. RFE is an effective gene selection algorithm that fits a diagnostic model recursively and removes weakest gene features per iteration until a specific optimal number of gene features is selected, while attempts to eliminate collinearity among gene features in the model^25^. The genes are ranked by gene importance of the model^25^. Logistic regression with LASSO penalty is gene-selection method, which uses regularization parameter to shrink insignificant regression coefficients to zero and this method will automatically select those genes that are useful, discarding redundant or non-informative genes in asthma prediction^26^. The Boruta algorithm uses a random replicate of the original data to create shuffled copies of all features which are called shadow features. Then, the algorithm performs a classification matrix using all features to compute the most important features. The shadows’ feature importance is used as a reference for evaluating the scores obtained by the actual features^27^. The potential features yielded the ‘‘confirmed’’ status in Boruta iterations and achieved higher importance than the best shadow was selected. Boruta algorithm is an extended version of RF and widely used for selecting gene-signature associated with response variable^28,29^. Despite each method has its own strength, there are limited studies that examine which method perform better in risk prediction including asthma; particularly when there is high correlation among gene features. The four methods were used to screen potential gene-features and their asthma classification performance were compared.

### Construction of asthma classification models and validation

To compare which method outperforms in classifying asthmatic from control subjects based on the same number of gene-signatures obtained from the four methods (LASSO, RF, RFE, and Boruta) in multiple tissue/cell types, two broadly used classifiers RF and SVM were selected. RF algorithm was used for both feature selection/prioritization and classification^24,25^. We also used SVM algorithm as classifier to evaluate classification performance of the identified gene-signatures based on different models in multiple tissue/cell types^30,31^. The SVM and RF algorithms were used to predict asthma in the discovery sets: NECs and AECs tissue/cell types and validation sets: BECs, ASM and WB tissue/cell types and finally the best risk prediction method for different tissue/cell type datasets in discriminating asthmatic from control subjects was selected.

### Evaluating classification performance

The model diagnostic performance of different feature selection and classification methods were evaluated based on different performance metrics including AUC, Matthew's correlation coefficient (MCC), and F1-score (F-measure). Multiple model prediction performance metrics were used to avoid overoptimistic results when the number of asthmatic and control subjects are unbalanced, as previous studies suggested^32,33^. The AUC of ROC curve is the approximation of the area under precision-recall curve, whereas F1-score and MCC are defined as follow.

$${F}_{1}=\frac{TP}{TP+\frac{1}{2}(FP+FN)}$$

$$MCC=\frac{TP\times TN-FP\times FN}{\sqrt{(TP+FP)(TP+FN)(TN+FP)(TN+FN)}}$$

where, TP, TN, FP and FN represent the number of correctly predicted asthma class, the number of correctly predicted control class, the number of incorrectly predicted asthma class and the number of incorrectly predicted control class, respectively. F1 equal to 1 shows perfect model classification performance and 0 implies the model is imperfect. MCC values range from –1 to 1, the model classification performance is perfect at 1 and completely incorrect classification at -1.

### Functional annotation and enrichment analysis

To identify the biological function underlying differentially co-expressed genes in each significant asthma associated module, we performed pathway enrichment analysis by Ingenuity Pathway Analysis (IPA) software (http://www.ingenuity.com/products/ipa). The IPA method evaluates proportional representation of module genes from a defined set in a canonical pathway in all set of known genes. Canonical pathways of the input module genes were evaluated to identify significantly enriched pathways adjusting for multiple testing. The *p*-value is calculated based on a right-tailed Fisher Exact test. For canonical pathway analysis, a ‑log (*P*‑value) > 2 was taken as threshold to define significant canonical pathways^34^.

## Results

### Identification of differentially expressed genes (DEGs) in asthma

**Figure 2:**
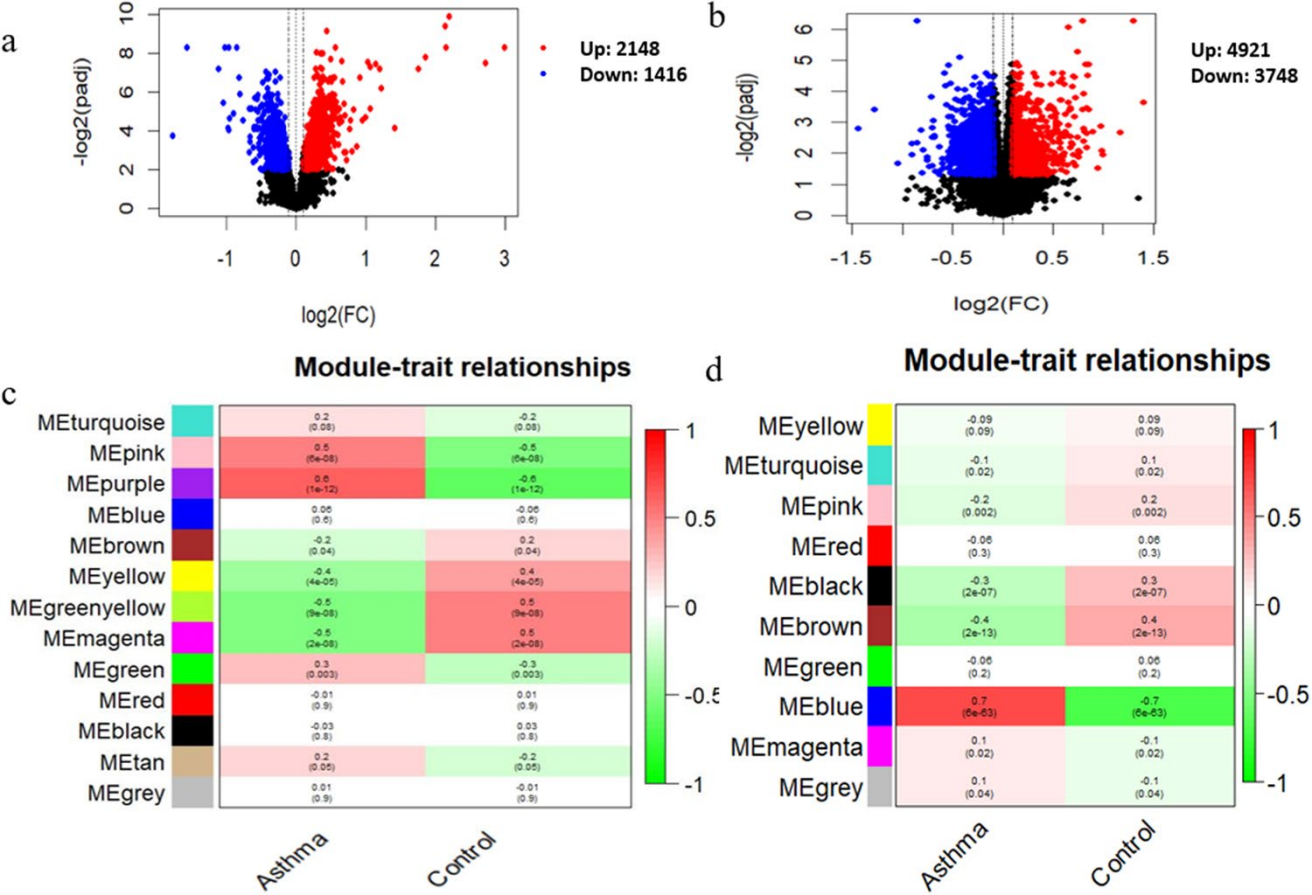
Identification of asthma related genes for the AECs and the NECs. (**a**, **b**) Volcano plot showing 3564 DEGs for the AECs and 8669 DEGs for NECs respectively. DEGs- differentially expressed genes (adjusted *p*-value < 0.05). (**c**) The correlation between 13 modules and asthma status in the AECs data. The modules associated with asthma include purple module, magenta module, pink module, greenyellow module, yellow module, green module and brown module in the AECs dataset. (**d**) The correlation between 10 modules and asthma status in the NECs data. The modules associated with asthma include blue module, brown module, pink module and black module in the NECs dataset. We kept genes within the selected modules for each dataset for subsequent analysis.

The genome wide DEG analysis results for asthma in the AECs and NECs were visualized via volcano plot (Fig. 2a,b). The results showed that a total of 3564 genes from AECs in Fig. 2a and 8669 genes from NECs data in Fig. 2b were differentially expressed with adjusted *p-value* < 0.05, and these DEGs were retained for subsequent analysis.

### Identification of asthma associated key modules and co-expressed genes

To characterize the correlation structure of 5833 and 7496 hypervariable genes and further examine their gene-regulatory networks in AECs and NECs, respectively, we conducted WGCNA analysis using hierarchical agglomerative clustering with average linkage. For AECs dataset, the suitable soft threshold power (β) = 8 (scale-free ${R}^{2}$ = 0.9) was used as the correlation coefficient threshold to ensure relatively balanced mean connectivity and scale free network (Fig. S1a). WCGNA revealed a total of 13 modules in the AECs dataset (Figs. S2a and 2c). For NECs dataset, power (β) = 5 (scale-free R^2^ = 0.86) was used as the correlation coefficient threshold to ensure balanced connectivity and scale free network (Fig. S1b) and the result of WGCNA analysis showed a total of 10 modules in the NECs dataset (Figs. S2b and 2d). To identify module-trait association, the estimated eigengenes values were correlated with the clinical traits of asthmatic and control subjects in the AECs and NECs datasets as indicated in the heatmap (Fig. 2c,d; |r| $\ge$ 0.2 and *P*-value < 0.001). Seven modules (purple, pink, greenyellow, brown, magenta, yellow, and green) in AECs and four modules (blue, brown, pink and black) in NECs were identified as significantly correlated with asthmatic subjects. The purple, pink and green modules were positively correlated with asthmatic subjects, while the brown, yellow, greenyellow and magenta were negatively correlated with asthmatic subjects in AECs dataset. The blue module was positively correlated with asthmatic subjects, while the brown, pink and black modules were negatively correlated with asthmatic subjects in NECs dataset. A total of 2495 co-expressed genes were found in seven significant modules including purple module (170 genes), magenta module (244 genes), pink module (283 genes), greenyellow module (86 genes), yellow module (467 genes), green module (455 genes) and brown module (790 genes) in the AECs dataset (Table S1), while a total of 2634 co-expressed genes were found in four significant modules including blue module (1225), brown module (961), pink module (211) and black module (237 genes) in the NECs dataset (Table S1).

### Selection of DCEGs in asthma correlated modules

Next, overlapping analysis between 3564 DEGs and 2495 co-expressed genes in six asthma correlated modules derived from AECs dataset resulted a total of 854 DCEGs (Table S1). Similarly, overlapping analysis between 8669 DEGs and 2634 co-expressed genes in four asthma correlated modules derived from NEC data resulted a total of 725 DCEGs (Table S1). These identified DCEGs in both AECs and NECs dataset were used for functional enrichment and asthma diagnostic gene-signature based model development.

### Functional analysis of the DCEGs in asthma correlated modules

**Figure 3:**
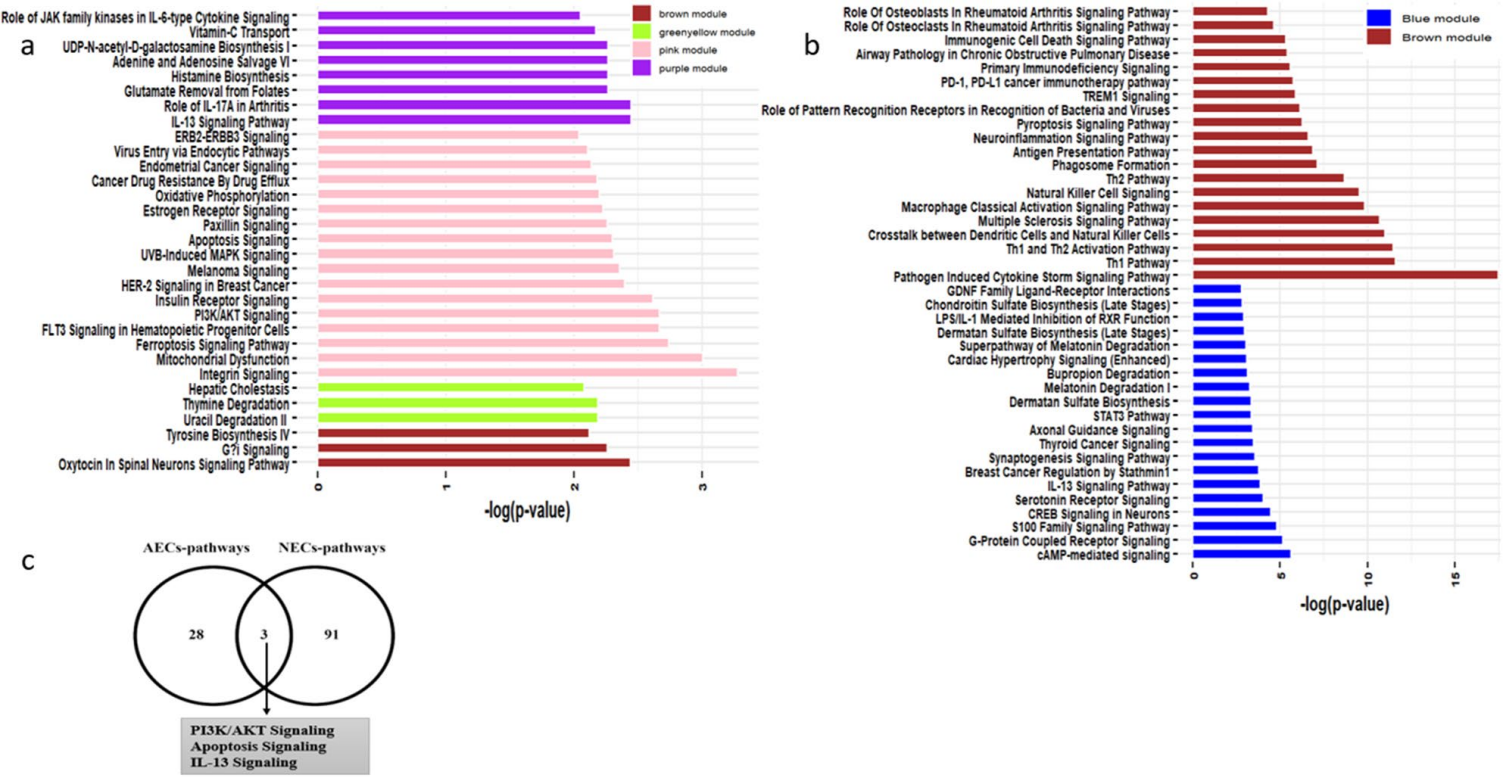
The significant canonical pathways of DCEGs associated with (**a**) purple module, pink module, greenyellow module, and brown module derived from AECs dataset (**b**) blue module and brown module derived from NECs dataset and (**c**) common canonical pathways of correlated genes derived from AECs and NECs datasets.

To obtain further insights into the biological function of the DCEGs in significant asthma associated modules derived from AECs and NECs datasets, the biological function enrichment analyses were performed using IPA software and the results are shown Fig. 3a,b and Tables S2, S3. The functional enrichment analysis of 132 unique DCEGs in the purple module derived from AECs dataset enriched in key biological functions such as IL-13 Signaling, role of IL-17A in arthritis, glutamate removal from folates, histamine biosynthesis (Fig. 3a). The enrichment analysis of 163 correlated genes in the pink module involved  in several biological functions, for example mitochondrial dysfunction, PI3K/AKT Signaling, and others (Fig. 3a). Other functional enrichment of correlated genes in asthma correlated modules (greenyellow and brown modules) derived from AECs dataset are shown in Fig. 3a and Table S2. Meanwhile, pathway analysis of DCEGs in the two most asthma correlated modules (blue and brown modules) derived from NECs dataset showed enrichment in several biological functions. The most enriched pathways of 1225 correlated genes for blue module included integrin signaling, CAMP-mediated signaling, protein coupled receptor signaling, S100 family signaling, IL-13 Signaling (Fig. 3b and Table S2). The 961 correlated genes in brown module involved in pathogen induced cytokine storm signaling, Th1 and Th2 Activation, crosstalk between dendritic cells and natural killer cells (Fig. 3b and Table S3). The functional enrichment overlapping analysis of correlated genes associated with asthma relevant modules of purple and pink modules derived from AECs and blue and brown modules derived from NECs were enriched in biological functions including IL-13 Signaling and PI3K/AKT signaling and apoptosis signaling (Fig. 3C and Tables S2, S3).

### Selection of potential genes associated with asthma

Based on the diagnostic gene selection methods discussed in material and method section, four ML-methods were applied to further select and prioritize asthma associated gene-signature in AECs dataset (n = 105) from the total of 854 DCEGs. Logistic regression with LASSO penalty and five-fold cross-validation was implemented to identify optimal $\lambda$ value = 0.02 which is derived from minimum binomial deviance, which was related to 30 DCEGs in predicting asthma in AECs dataset (Fig. S3a,b). Three other ML-algorithms including RF, Boruta and RFE were also used to prioritize and select top 30 DCEGs based on the relative importance of each DCEG in asthma prediction (Fig. S3c–e).

### Constructing gene expression-based asthma classifier models

**Figure 4:**
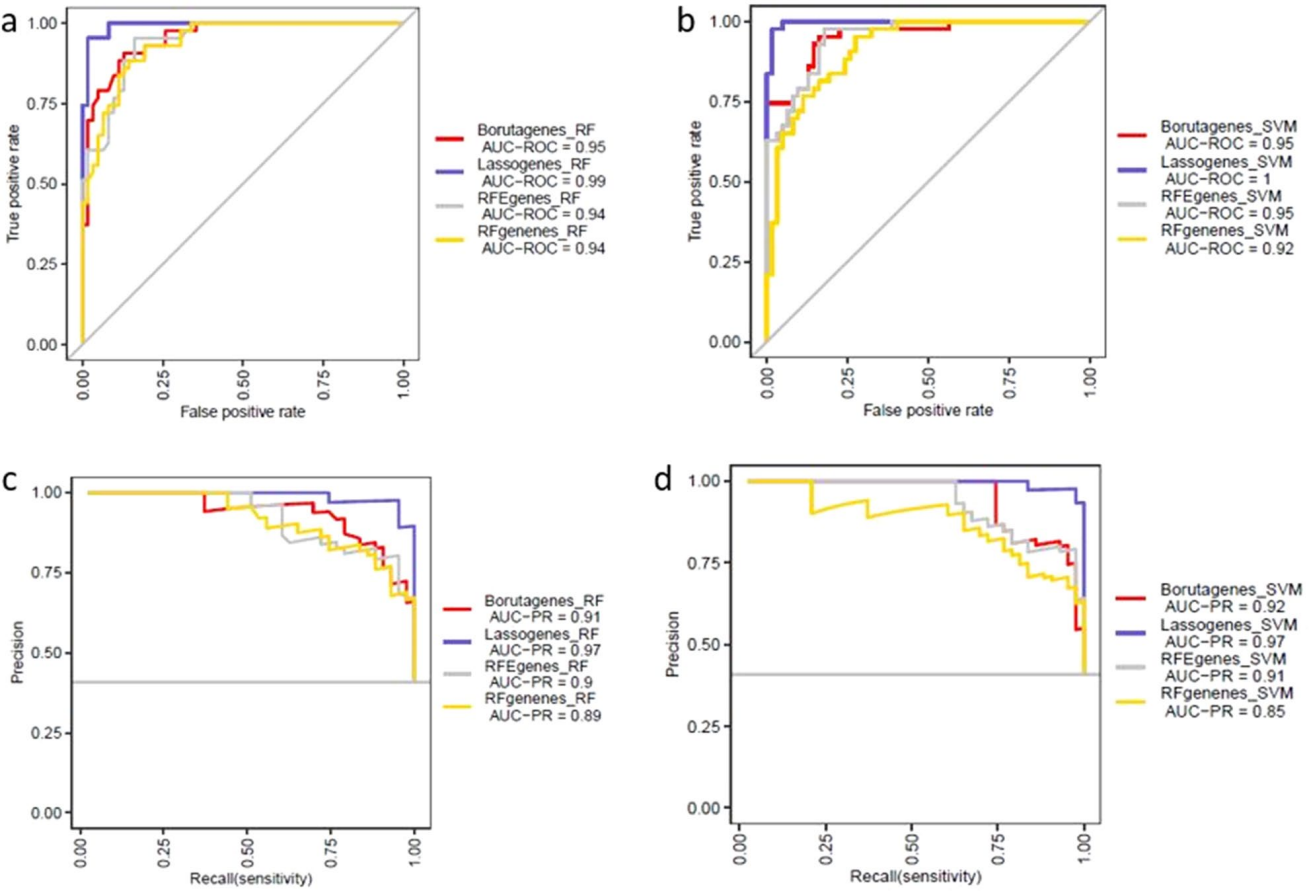
Model comparison of different gene selection methods (**a**, **b**) AUC values of different gene feature ranking methods-based RF and SVM classifiers in AECs dataset, respectively (**c**, **d**) AUC precision-recall (AUC-PR) curve values of different gene feature ranking methods based RF and SVM classifiers in AECs dataset, respectively.

To compare the power of discrimination between asthmatic and control subjects, we examined 30-gene signature identified by distinct ML feature selection algorithms: LASSO, RF, RFE and Boruta. The diagnostic performance of selected genes by four methods are shown in Fig. 4a–d in AECs dataset. The diagnostic ability of LASSO using 30-gene signature and AECs dataset showed AUC = 0.99 and AUC = 1 based on RF and SVM classifiers, respectively (Fig. 4a,b). LASSO-based genes performed better compared with other methods in discriminating asthmatic from control subjects in AECs dataset. Moreover, the AUC precision-recall curve (AUC-PR) was used as additional measure of model to control potential misleading of AUC curve. Importantly, AUC-PR measure of LASSO selected genes showed superior ability in classifying asthmatic from control subjects (Fig. 4c,d). Furthermore, the sensitivity, specificity, MCC and F-score values of LASSO gene selection method revealed better performance in classifying asthmatic from control subjects in AECs dataset (Fig. S3f and Table S4).

**Figure 5:**
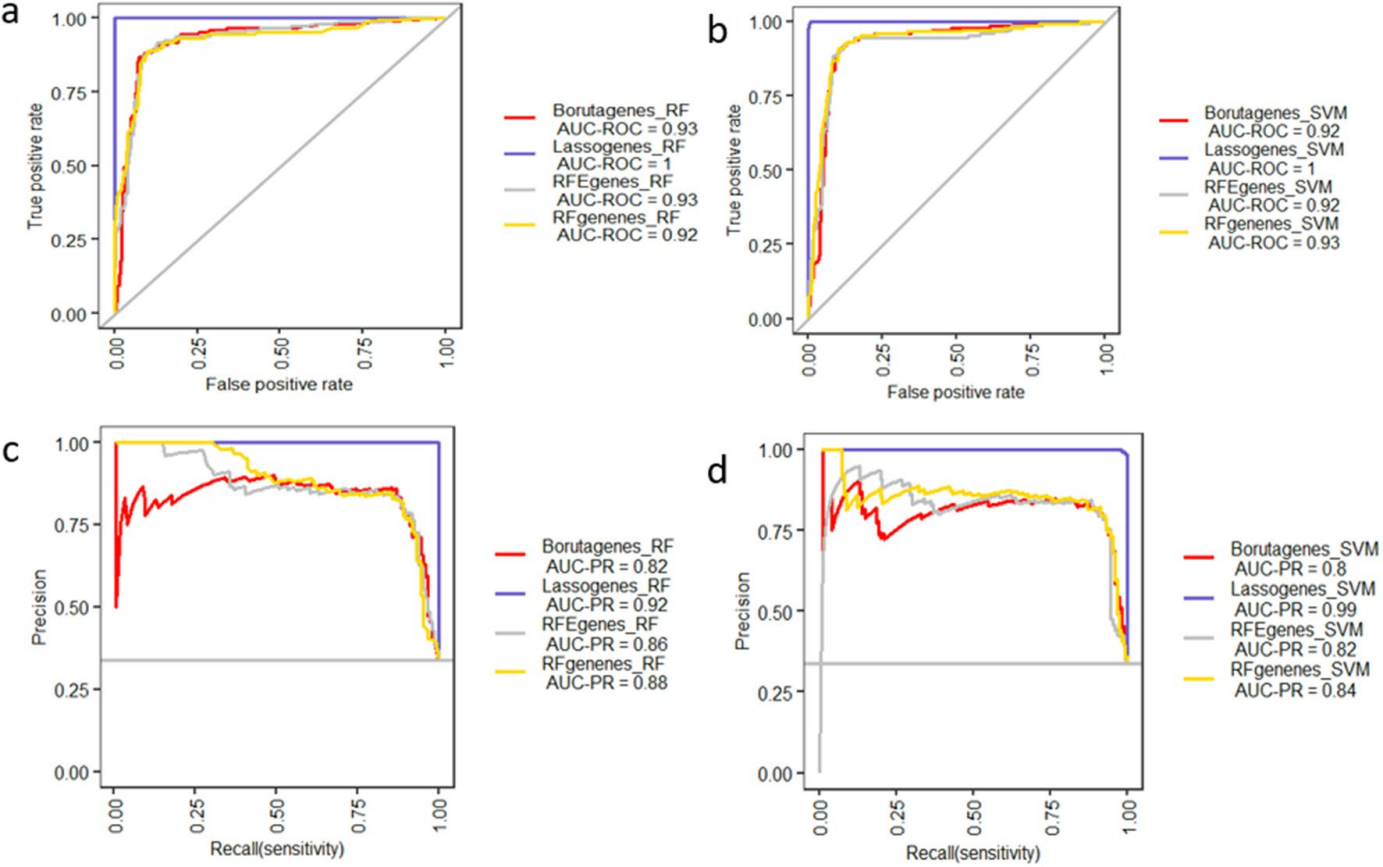
Model comparison of different gene selection methods (**a**, **b**) AUC values of different gene feature ranking methods-based RF and SVM classifiers in NECs dataset, respectively (**c**, **d**) AUC precision-recall (AUC-PR) curve values of different gene feature ranking methods based RF and SVM classifiers in NECs dataset, respectively.

The methods to construct diagnostic model, evaluate and validate for asthma prediction in NECs dataset, are analogues to the diagnostic model in AECs dataset. LASSO method with tenfold cross-validation identified 34 DCEGs in predicting asthma in NECs dataset (Fig. S4a,b) with optimal λ value = 0.004. Moreover, three methods (RF, Boruta and RFE) were used to prioritize and select top 34 potential gene signatures based on the relative importance of each DCEG in asthma prediction. The corresponding results are shown in Fig. S4c–e. Next, the diagnostic performance of four methods were examined using RF and SVM classifiers and their corresponding results are indicated in Fig. 5a–d. Notably, the diagnostic performance of LASSO identified 34-gene signature based on RF (AUC = 1) and SVM (AUC = 1) classifiers showed higher diagnostic performance in classifying asthmatic subjects from controls in NECs dataset (Fig. 5a,b). In addition, the AUC-PR values indicated that LASSO method with RF (AUR-PR = 0.92 and SVM (AUC-PR = 0.99) classifiers revealed in superior ability for classifying asthmatic subjects from control compared with other methods (Fig. 5c,d). Furthermore, the specificity, MCC and F-score values of LASSO with SVM classifier showed that the LASSO method had better classifying ability compared with other methods in NECs dataset (Fig. S4d and Table S5).

### Evaluation of the diagnostic models using independent data

**Figure 6:**
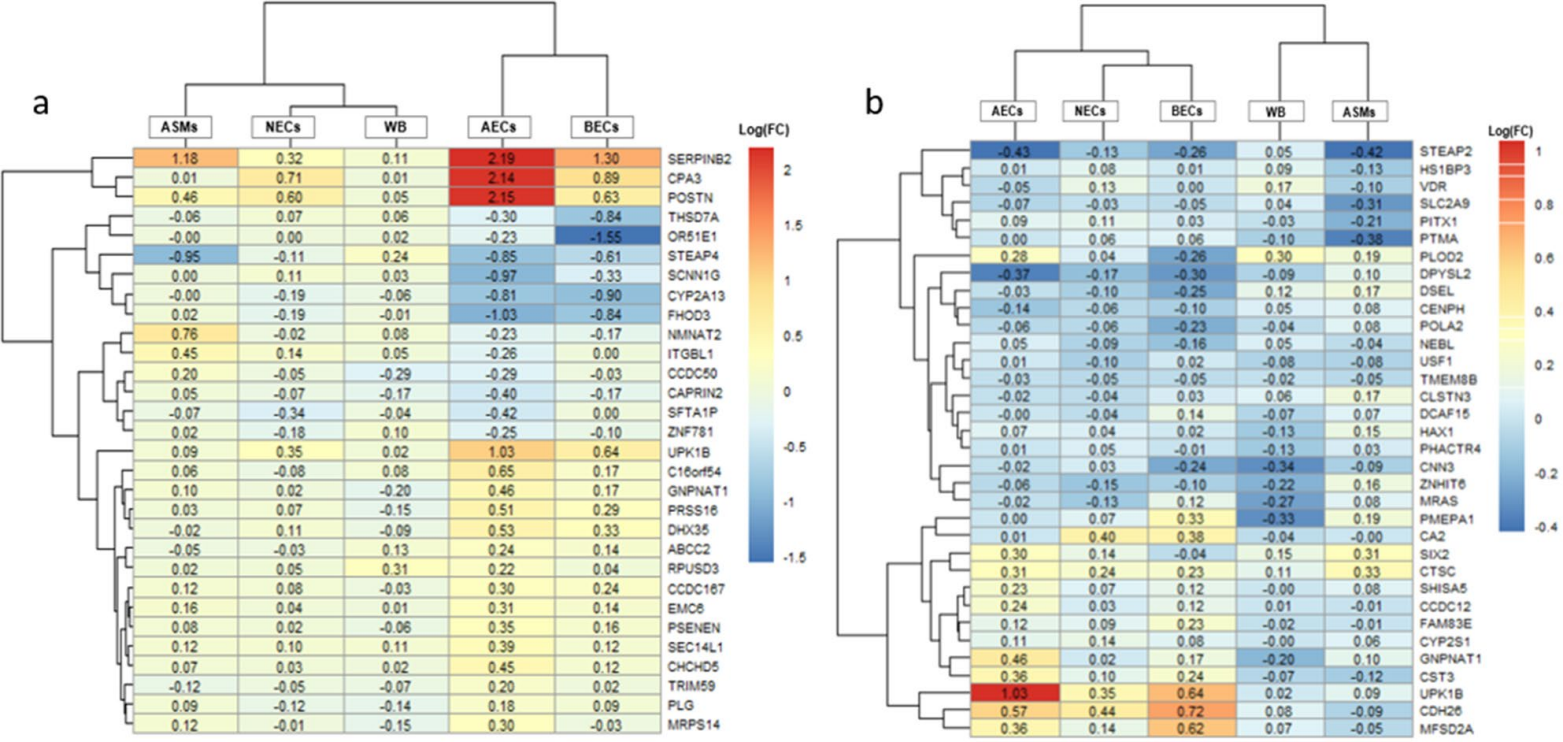
The heatmap showing the multiple tissue/cell type datasets logFC distribution of the gene signatures derived from AECs and NECs datasets. (**a**) 30-gene signature in various tissue types including NECs: nasal epithelial cells, AECs: airway epithelial cells, ASM: airway smooth Muscle and BECs: bronchial epithelial cells and WB: whole blood cells. (**b**) 34-gene signature in NECs, AECs, ASMs, BECs, and WB cells. Log2 fold-change is the log2-ratio of (expression in asthmatic subjects/expression in control subjects).Upregulation and downregulation in asthmatic compared with control subjects are reflected by log2 FC > 0 and < 0, respectively. FC = fold-change. The heat map of multiple tissue/cell type datasets of log FC values of gene signatures were generated using the pheatmap Version: 1.0.12 package ((https://cran.r-project.org/web/packages/pheatmap/index.html) in R.

To evaluate and compare whether the 30 and 34 gene-signatures derived from AECs and NECs datasets perform well in distinguishing asthmatic subjects from controls, various tissue/cell types of datasets including BECs, ASM and WB tissue/cell types were used as model validation datasets. Initially, the differential co-expressed AEC-and NCE-derived gene signatures between asthmatic subjects and controls were compared in validation datasets. Notably, the identified gene signatures-derived from AECs and NECs were found to be expressed in all three validation datasets (Fig. 6a,b), where nine genes including CPA3, SERPINB2, CHCHD5, EMC6, RPUSD3, POSTN, SEC14L1 and UPK1B derived from AECs dataset were persistently upregulated in asthmatics subjects compared with controls. Out of 34 gene-signatures derived from NECs, two genes (CTSC and UPK1B) were persistently upregulated while one gene TMEM8B were persistently downregulated in asthmatic subjects compare with controls in all validation datasets. Other gene-signatures showed tissue specific differential expression.

**Figure 7:**
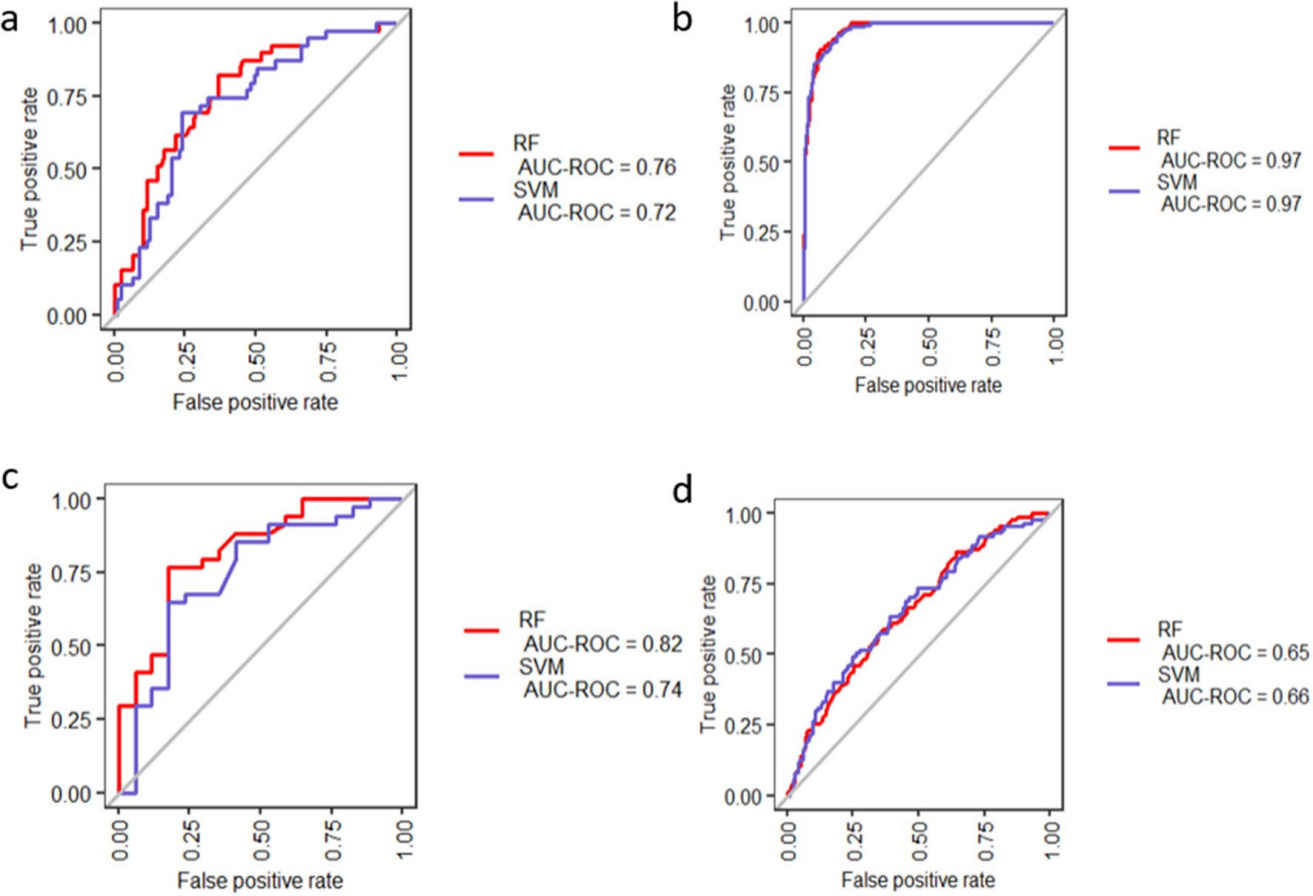
Validation of the 30-gene-signature based diagnostic model derived from AEC data. The classification performance is presented in terms of AUC values based RF and SVM methods in discriminating asthmatic subjects from control for (**a**) BECs, (**b**) NECs, (**c**) ASM, and (**d**) WB datasets.

After evaluating the differential expression of 30 and 34 gene-signatures, we examined their diagnostic performance in various cell/tissue types. The diagnostic performance of 30-gene signature-based RF and SVM classifier algorithms are shown in Fig. 7. Using SVM classifier with 30-gene-signture-derived from AECs data, the AUC values achieved were 0.72, 0.97, 0.74 and 0.66 in BECs, NECs, ASM and WB, respectively (Fig. 7a–d). Using RF classifier, AEC-derived gene-signature, the AUC values for the four validation datasets were 0.76, 0.97, 0.82 and 0.65, respectively (Fig. 7a–d). For RF classifier, the AUC-PR values in the in BECs, NECs, ASM and WB were equals 0.57, 0.94, 0.87 and 0.31, respectively (Fig. S5a-d). Moreover, model performance measures including sensitivity, specificity, MCC and F1-score of the 30-gene signature-based model derived from AEC data are shown in Fig. S5e and Table S6. Using SVM classifier in BECs, NECs, ASM and WB datasets, 30 gene-signature based diagnostic model derived from AECs exhibited a performance with MCC of 0.44, 0.79, 0.44 and 0.24, respectively (Table S6 and Fig. S5e). Furthermore, 30-gene signature using SVM classifier in BECs, NECs, ASMs and WB datasets exhibited a performance with F1-score of 0.64, 0.86, 0.85 and 0.41, respectively (Table S6). The results showed that AECs-derived diagnostic model had better classification performance in the BECs, NECs and ASM data sets. Relatively, diagnostic model showed lower classification ability when the model was tested on WB dataset.

**Figure 8:**
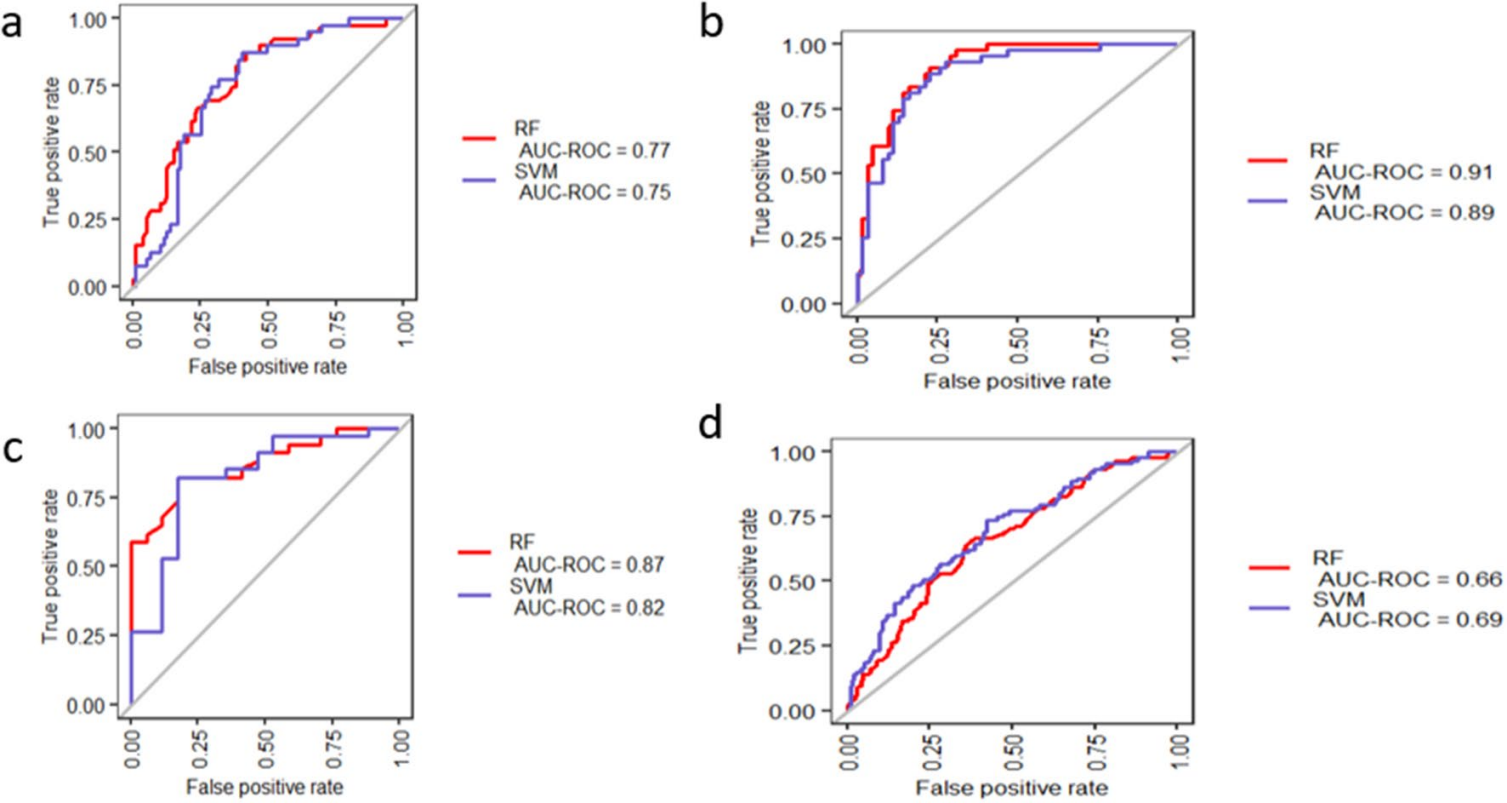
Validation of the 34-gene-signature based diagnostic model derived from NEC dataset. The classification performance is presented in terms of AUC values based RF and SVM methods in discriminating asthmatic subjects from control for (**a**) BECs, (**b**) NECs, (**c**) ASM and (**d**) WB datasets.

Similarly, the diagnostic performance of model derived from NECs data showed that the AUC value of SVM classifiers were 0.75, 0.89, 0.82 and 0.69 in BECs, AECs, ASM and WB, respectively (Fig. 8a–d). The diagnostic performance of model derived from NECs data showed that the AUC values based on RF classifier equals to 0.77, 0.91, 0.87 and 0.66 in BECs, AECs, ASM and WB, respectively (Fig. 8a–d). The AUC-PR values of SVM classifier in the in BECs, NECs, ASM and WB attained 0.57, 0.83, 0.88 and 0.32 to respectively (Fig. S6a–d). Moreover, the sensitivity, specificity, MCC and F1-score of the 34-gene signature-based model derived from NECs data are shown in Fig. S6e and Table S6. As indicated in Fig. S6e and Table S6, the 34-gene signature using SVM classifier was tested in BECs, AECs, ASM and WB validation sets and the model performance of MCC value for each dataset was equal to 0.44, 0.65, 0.63, and 0.26, respectively. The diagnostic model showed a performance with F1-score value of 0.65, 0.80, 0.86 and 0.44 in the BECs, AECs, ASM and WB validation sets, respectively. The diagnostic model derived from NEC dataset also indicated that model perform well in the BECs, AECs and ASM compared with WB validation set.

## Discussion

In our study, we developed diagnostic models based on asthma associated gene signatures obtained from a total of 105 AECs and 393 NECs subjects and validated in various tissue/cell types. We performed an integrated analysis of  differential gene expression analysis,WGCNA and machine learning to identify potential gene signatures that discriminate asthmatic subjects from controls. Frist, we identified 854 and 725 asthma associated DCEGs in AECs and NECs datasets, respectively based integrated analysis of DEGs and WGCNA methods. Then, four machine learning algorithms including LASSO, RF, RFE and Boruta methods were used to select potential asthma associated DCEGs and their discriminating power and model performance measures were evaluated in both AECs and NECs datasets. The results showed that LASSO method identified 30 and 34 gene-signatures and showed better asthma prediction performance in AECs and NECs datasets, respectively. The validation datasets in independent multiple tissue/cells suggested that gene-signatures-derived from nasal/upper airways epithelium gene signature-based model could distinguish asthmatic subjects from controls in multiple tissue/cell types including BECs, ASMs and WB cells. The results suggested that the identified gene-signatures may be serve as promising a minimally invasive biomarker for asthma diagnosis.

Despite it is ideal to develop gene-signature based model-derived to obtain samples from target tissues in diseases development (e.g. from lung tissue), it is not feasible and difficult specifically when a large sample size is needed for developing diagnostic tools with robust statistical power. Similar to previous studies, our asthma diagnostic classifiers were developed based on surrogate cell/tissue types and target cell/tissue^9,10,35^. An experimental study suggested to use nasal epithelial cells as surrogate for bronchial epithelium cells for asthma^10^. Despite several previous studies developed classification models to predict asthma, most of the studies focused on gene expression data from single tissue^11,36^. Previous study compared different tissue types including AECs, NECs and peripheral blood mononuclear cells to predict asthma using DNA methylation data and showed that asthma diagnostic model derived from AECs and NECs tissue/cell types resulted better asthma prediction performance compared with peripheral blood mononuclear cells^37^.

To characterize and understand DCEGs and their functional enrichment, WGCNA analysis was used in tissues that are quite related, which increases the expectation that a gene network signature in a tissue like NECs will replicate in AECs. Four modules (purple, pink, greenyellow and brown) in AECs and three modules (blue, brown and pink) in NECs were identified as significantly correlated with asthmatic subjects. The purple and pink modules were positively correlated with asthmatic subjects in AECs. The blue and brown were positively and negatively correlated with asthmatic subjects, respectively in NECs. We showed that DCEGs within asthma associated modules in AECs and NECs datasets were correlated with the expression of different genes that revealed distinct biological signaling and harbored gene-network signature associated with asthmatic subjects. Asthma associated pink and purple modules in AECs and blue and brown modules in NECs, and associated pathways showed an overlapped asthma related pathways including IL-13 Signaling and PI3K/AKT Signaling and apoptosis signaling. Notably, AEC and NEC-derived correlated gene signatures including CCL26, CLCA1 and POSTN were involved in IL-13 signaling, where IL-13 signaling of the airway epithelium is associated pathophysiology of asthma and airway inflammation^38^. The purple asthma associated module derived from AECs were enriched in mitochondrial dysfunction and PI3K/AKT signaling. The DCEGs uniquely correlated with asthma associated brown module derived from NECs was enriched in pathogen induced cytokine storm signaling, Th1 and Th2 Activation, crosstalk between dendritic cells and natural killer cells that suggests potential mechanism among these enriched pathways. Overall, our findings showed that NECs and AECs derived DCEGs enriched in asthma related pathways that may drive asthma pathology. Next, considering DCEGs derived from AECs and NECs datasets as candidate features, we used four ML methods to select potential gene-signatures that were important for subsequent validation.

ML methods were used to develop asthma diagnostic model in predicting asthmatic subjects from controls^17^. However, relatively limited studies were focused integrating analysis of DEGs, WGCNA and ML methods in asthma prediction. In this study, different models comparison showed that DEGs, WGCNA followed by LASSO method identified 30 and 34 potential gene signatures, respectively. In AECs and NECs datasets with higher performance in discriminating asthmatic subjects from controls. Several previous transcriptomics studies used DEGs and ML approach to construct diagnostic and or prognostic models^39,40^. We compared our approach with standard combined DEG + ML approach and the result showed similar performance (Supplementary Fig. S7 and Supplementary Table S7). However, integrated analysis of DEG, WGCNA and ML approach is essential approach to select key co-expressed genes for the exploration of biological function, pathway, etc. and also to alleviate multiple testing problem by reducing feature size and hence minimize computational cost compared with combined analysis of DEG and ML approach^41^. Hence, we implemented DEG + WGCNA + LASSO model to select candidate genes for downstream analyses and validation.

LASSO identified potential DCEGs includes CPA3, SERPINB2, CHCHD5, EMC6, RPUSD3, POSTN, SEC14L1 and UPK1B in AECs dataset and these DCEGs were also persistently upregulated in multiple tissue/cell type datasets from asthmatic subjects. The previous study reported that elevated expression of CPA3 gene was observed in asthmatic subjects compared to controls and CPA3 gene correlated with sputum mast cells, asthma and rhinitis^42^. Recent study reported that the expression level of SERPINB2 gene was increased in airway epithelial cells of asthmatic and in atopic asthmatic subjects compared controls^43^.

The LASSO identified five potential DCEGs in NECs dataset includes SIX2, CDH26, NEBL, CTSC and SLC2A9A. Several DCEGs identified in this study demonstrated biological function relevant to asthma. For example, a previous study showed that abnormality CDH26 gene are characterized by IL-13 stimulation of the airway epithelium and T2 inflammation of the airway epithelium in asthma development^44^. Yang et al. (2017) reported that CTSC gene was elevated in asthmatic subjects, which was also associated with methylation marks of subjects with asthmatic and allergy^45^. It has been reported that CTSC gene is maturated by a multistep proteolytic process and is secreted by activated cells during inflammatory lung diseases^46^. Our study also confirmed that CTSC gene was not only upregulated and co-expressed with other potential asthma related genes in nasal epithelium of asthmatic subjects but also persistently upregulated in multiple tissue/cell types of asthmatic subjects, which reflects that upregulation of CTSC gene in multiple tissue/cell may have functional association with the development and progression asthma disease.

To the best of our knowledge, our study is one of the first to develop asthma diagnostic models using differential expression analysis and co-expression network combined with machine learning based on microarray and RNAseq datasets of AECs and NECs tissue/cell sample types. Prioritizing and identifying potential gene signatures to construct asthma diagnostic model from easily accessible tissue/cell types are vital to elucidate pathological process of asthma at molecular level, and to extend adequate evidence for the development of therapeutic target. The main contribution of the current study is to identify potential gene signatures and to compare diagnostic performance of different machine learning methods in classifying asthmatic from control subjects based on AECs and NECs tissue/cell datasets and validate the diagnostic models, which are stable and show robust performance in classifying asthmatic from control subjects. Our method prioritized and identified potential asthma associated DCEGs, suggests several of which are implicated in asthma pathology.

More recently, machine learning and statistical methods haven been commonly used in RNA-seq and microarray data analysis of biomedical studies^47,48^. However, the analysis of high-dimensional genomic data has a number of challenges including model overfitting and multicollinearity problems (e.g., existence of DCEGs in modules). To address such problems, appropriate statistical machine learning methods are required. Here, to identify the appropriate gene selection method in distinguishing asthmatic subjects from controls, we evaluated different gene selection methods based on the results of DEGs and WGCNA in the derivation datasets and independent validation datasets. From classification performance, LASSO algorithm was identified as robust method to select potential gene signature to improve the diagnostic performance. Notably, all methods showed better diagnostic performance in the derivation sets. However, the robustness of the model should be validated in external validation datasets. In our study, we developed gene signatures based diagnostic models using NECs and AECs datasets, and validated to examine whether they can perform well in external datasets with different tissue/cell types including BECs, ASM cells and WB datasets. Moreover, we examined whether diagnostic models that derived from easily accessible cell/ tissues (NECs and AECs) can also serve as robust surrogate model for target cell/tissue (e.g., ASM cells, BECs) and easily accessible cells (e.g. WB cells) regardless of sequencing technology (microarray or RNA-seq). Notably, gene signature based diagnostic model derived from microarray gene expression AECs dataset and gene signature based diagnostic model derived from RNA-seq NECs dataset were validated and the analysis indicated that both diagnostic models showed a better performance in the BECs and ASM dataset compared with WB dataset. The reason could be gene expression derived from WB tissue may not specific to asthma conditions. Whereas validation of diagnostic model based on gene expression comes from the target tissue sources-BECs and ASM tissue/cell types showed better performance, where these target tissue/cell types have well known role in asthma exacerbations and airways remodeling^7,49^. Overall, the results showed that diagnostic models derived from NECs and AECs datasets can serve as surrogate source of biological samples for hard-to-get tissues including BECs dataset.

Most models perform better prediction in training dataset but predict poorly in external validation dataset^50^, may be due to overfitting problem. The best model should have high AUC, F1-score and MCC values^32^. Our gene-signature based diagnostic models derived from AECs and NECs data showed higher accuracy and stable performance in external different tissue/cell type datasets. The multiple tissue/cell validation datasets circumvent overoptimistic results and assure general reproducibility. Despite our developed diagnostic models showed promising performance in predicting asthma, the current study has still some limitations. Since this study focused on computational analysis based on retrospective samples, future validation of the identified signatures should be performed with functional experiments. The sample size in some public dataset is small, which may hide potential correlations between gene expression signatures and outcome variable. Future study should consider increasing sample size and other feature selection strategies to improve diagnostic prediction performance of asthma and other airway diseases.

In conclusion, we identified small number of differentially co-expressed gene signatures and established diagnostic models based on an integrated analysis of bioinformatics and machine learning methods to predict asthma diagnosis using airway epithelium gene expression data. Based on multiple-diagnostic performance criteria, we found that comparable diagnostic performance between AECs and NECs, which highlight the importance of gene-signature –based diagnostic models derived from AECs and NECs data as suitable surrogate model in predicting asthma diagnosis. More importantly, our diagnostic models are promising tool to improve decision making, which may provide potential gene signatures for diagnosis of asthma and other airway diseases.

## Supplementary Information

Supplementary Figures.

Supplementary Tables.

## Data Availability

All gene expression datasets supporting this work are freely accessible at NCBI GEO (https://www.ncbi.nlm.nih.gov/geo/) with accession numbers GSE67472, GSE152004, GSE69683, GSE201955 and GSE58434.

## Abbreviations

AECs: Airway epithelial cells
ASM: Airway smooth muscle
AUC: Area under the receiver operating characteristic curve
BECs: Bronchial epithelial cells
CV: Coefficient of variation
DCEGs: Differentially co-expressed genes
DEGs: Differentially expressed genes
LASSO: Least absolute shrinkage and selection operator
MCC: Matthews correlation coefficient
NECs: Nasal epithelial cells
RF: Random forest
RFE: Recursive feature elimination
ROC: Receiver operating characteristic
SVM: Support-vector machine
TOM: Topological overlap matrix
WB: Whole blood
WGCNA: Weighted gene co-expression network analysis

## Code availability

The R code for all analyses in this manuscript has been deposited as open-source code in GitHub at https://github.com/Mershalab-asthma/ADM.
