# Supplementary material for: Development and validation of asthma risk prediction models using co-expression gene modules and machine learning methods
Source: Sci Rep. 2023 Jul 12;13:11279. doi: 10.1038/s41598-023-35866-2 (PMC10338542; doi:10.1038/s41598-023-35866-2)
Supplement: Supplementary file 1 — Supplementary Figures. [file 41598_2023_35866_MOESM1_ESM.docx]

**Development and validation of asthma risk prediction models using co-expression gene modules and machine learning methods**

Eskezeia Y. Dessie^1^, Yadu Gautam^1^, Lili Ding^1^, Mekibib Altaye^1^, Joseph Beyene^2^, Tesfaye B. Mersha^1^

^1^Department of Pediatrics, Cincinnati Children’s Hospital Medical Center, University of Cincinnati College of Medicine, Cincinnati, OH, USA, ^2^Department of Health Research Methods, Evidence, and Impact, McMaster University, Hamilton, Canada

**List of supplementary Figures**


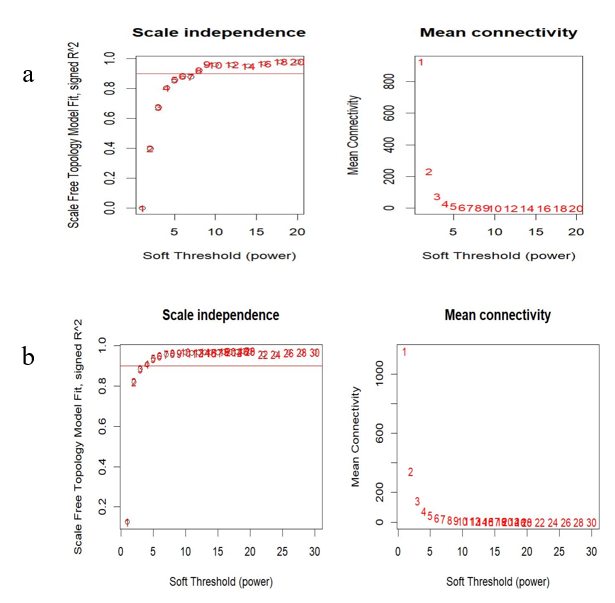


**Figure S1**: WGCNA analyses to select soft thresholding power for a) AECs dataset b) NECs dataset.


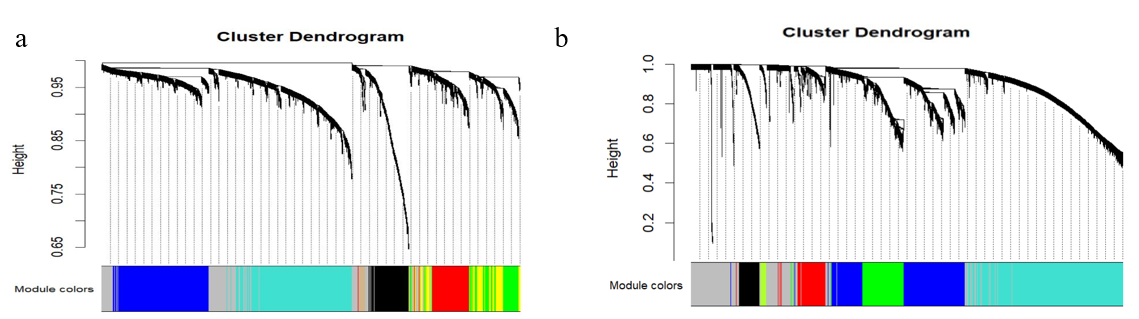


**Figure S2**: Cluster dendrogram of genes whose coefficient of variation (CV) > 4% in a) AECs dataset b) NECs dataset. The colors indicates co-expression modules and uncorrelated genes assigned to the gray module.


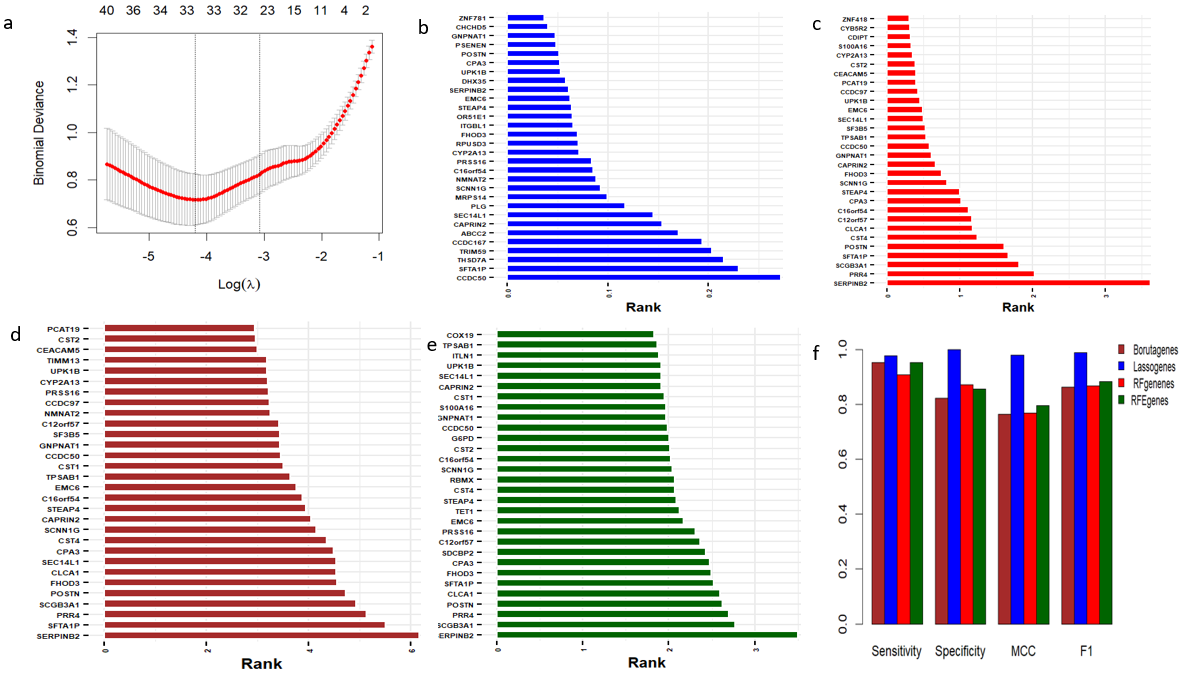


**Figure S3:** Potential DCEGs selected by four machine learning methods in the AECs dataset. a) Logistic regression with Lasso penalty alpha = 0.015 was used to select 30 important DCEGs. The bar plot showing prioritized and ranked DCEGs based on b) Lasso) c) RF d) Boruta and e) RFE f) Bar plot showing model comparison using various performance measures including sensitivity, specificity, MCC and F-score values 4 ML methods. RF-Random forest, Lasso-least absolute shrinkage and selection operator, RFE- Recursive Feature Elimination.


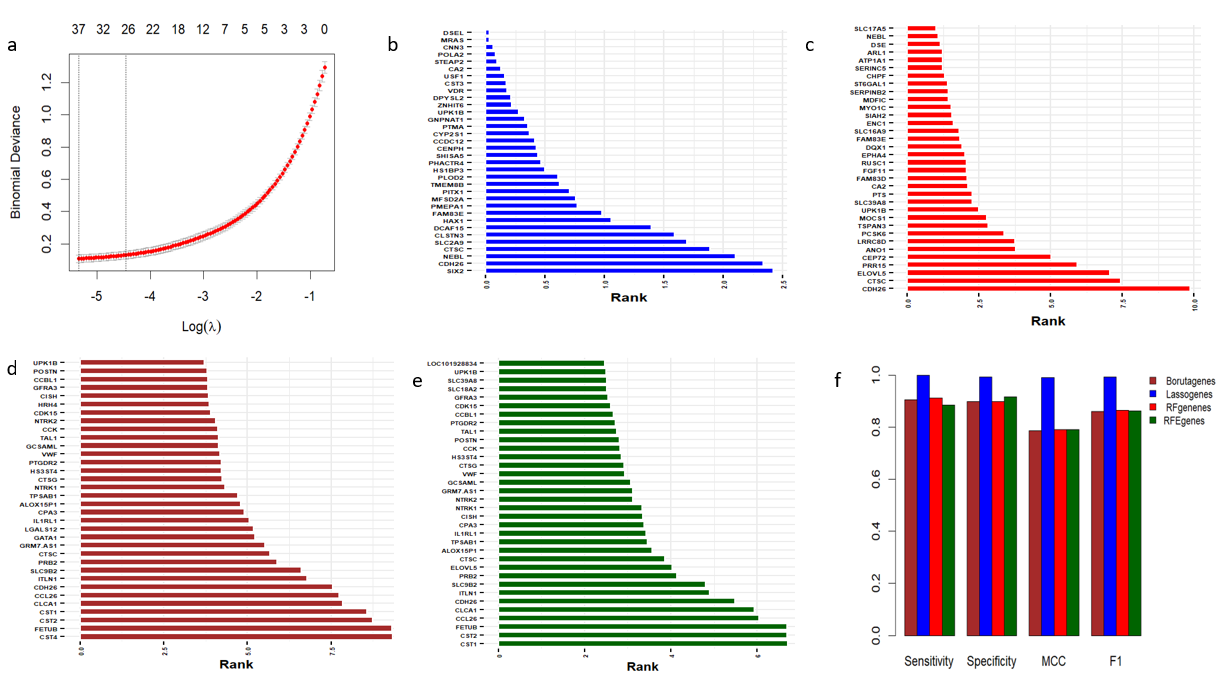


**Figure S4:** Potential DCEGs selected by four machine learning methods in the AECs dataset. a) Logistic regression with Lasso penalty alpha = 0.0048 was used to select 34 important DCEGs. The bar plot showing prioritized and ranked DCEGs based on b) Lasso) c) RF d) Boruta and e) RFE f) Bar plot showing model comparison using various performance measures including sensitivity, specificity, MCC and F-score values 4 ML methods. RF-Random forest, Lasso-least absolute shrinkage and selection operator, RFE- Recursive Feature Elimination.


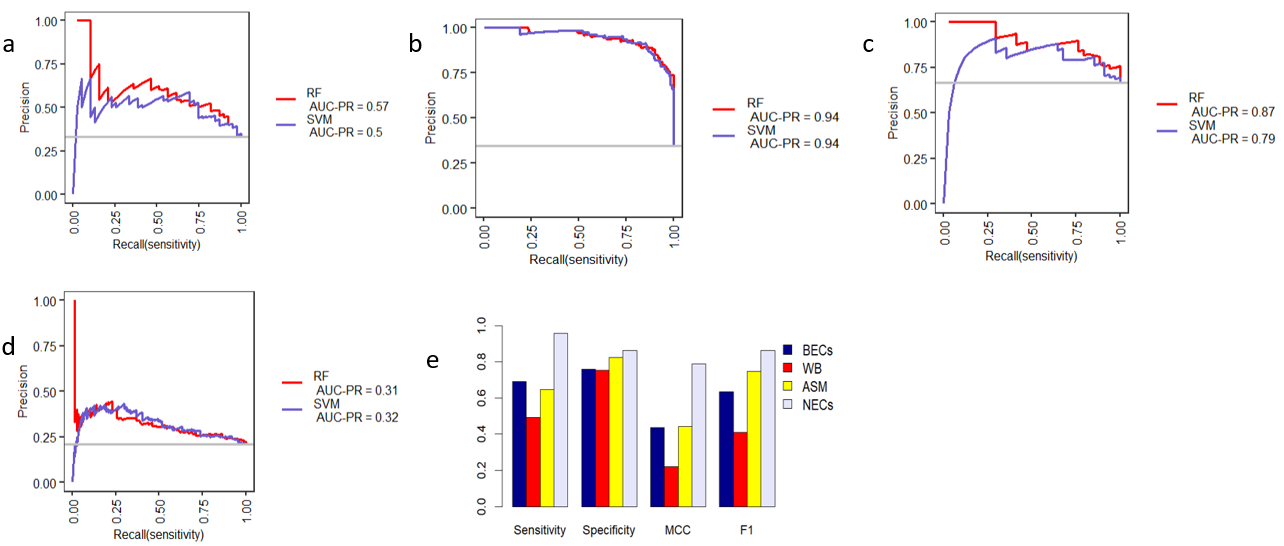


**Figure S5**. Evaluation of the 30-gene signature based diagnostic model derived from AEC dataset. The diagnostic performance is presented in terms of AUC the precision-recall curve ((AUC-PR) in a) BECs, b) NECs, C) ASM, d) WB datasets. e) Different diagnostic performance measures including sensitivity, specificity, MCC and AUC values in various cell/ tissue types including BECs, NECs, and ASM and WB datasets.


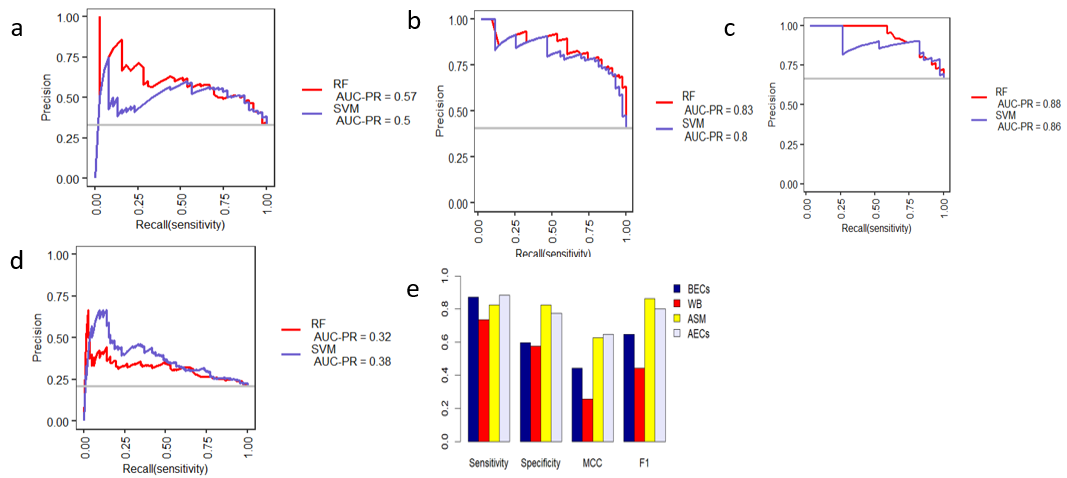


**Figure S6**. Evaluation of 34-gene signature based diagnostic model derived from NEC dataset. The diagnostic performance is presented in terms of AUC the precision-recall curve ((AUC-PR) in a) BECs, b) AECs, C) ASM, d) WB datasets. e) Different diagnostic performance measures including sensitivity, specificity, MCC and AUC values for various tissue types including BECs, AECs, and ASM and WB datasets.


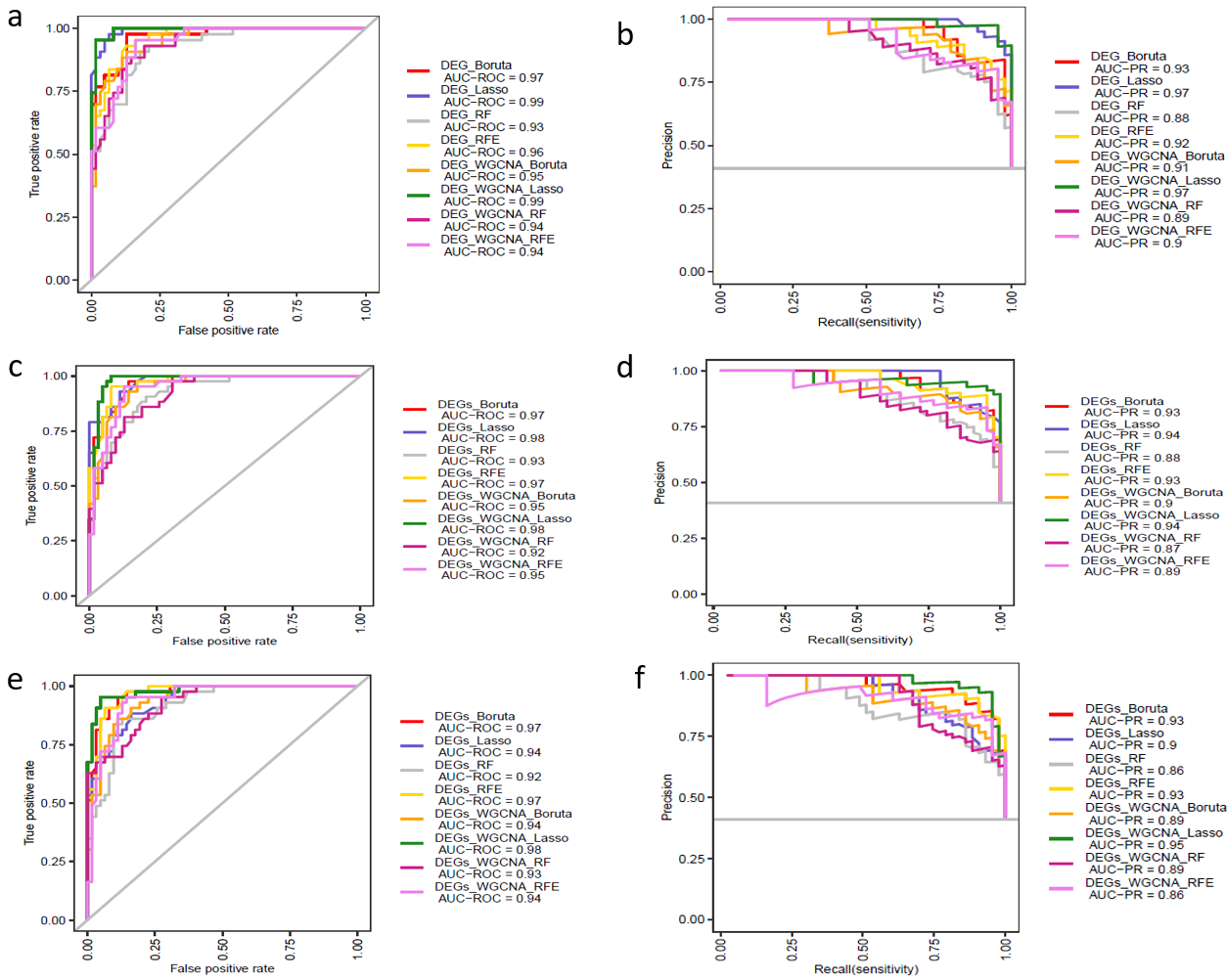


**Figure S7**. Comparison DEGs+ WGCNA+ML approach with the standard DEGs+ML approach.

The AUC values of top 30 prioritized genes obtained from different methods a) the precision-recall curve (AUC-PR) of 30 top prioritized genes b) The AUC values of top 20 prioritized genes obtained from different methods c) the precision-recall curve (AUC-PR) of 20 top prioritized genes c) The AUC values of top 10 prioritized genes obtained from different methods d) the precision-recall curve (AUC-PR) of 30 top prioritized genes.

To select and rank genes we followed the following steps. Initially, filtering based on DEGs (3564 DEGs) and filtering both DEGs and WGCNA methods (854 DCEGs) were performed. Then, we trained four different algorithms to prioritize and rank top 15, 20 and 30 genes from the list of DEG set ( n= 3564) based on the variable importance DEGs in model. Similarly, we also applied different machine learning method to rank 15, 20, and 30 prioritized genes from the list of DCEG set ( n = 854). We used 5-time repeated 5-fold CV to optimize our models. To compare different model performance, that is genes obtained from DEG+ML and DEG+WGCNA+ML approaches evaluated based on various performance measures including AUC precision-recall curve (AUC-PR), sensitivity, specificity, and MCC and F-score values. DEGs+WGCNA+Lasso and DEGs+ Lasso method showed relatively consistent higher performance in discriminating asthmatic subjects from controls. On the other hand, when we apply RFE and Boruta method, DEG+ML showed higher performance than DEG+WGCNA+ML approach. However, the computational cost large features for RFE method is too high. Overall, for our study we chose DEG+WGCNA+Lasso to reduce computational cost and identify biological relevant genes, and pathways. Hence, we implemented DEG+WGCNA+Lasso model to select candidate genes for downstream analyses and validation.
