# Supplementary material for: Development and validation of asthma risk prediction models using co-expression gene modules and machine learning methods
Source: Sci Rep. 2023 Jul 12;13:11279. doi: 10.1038/s41598-023-35866-2 (PMC10338542; doi:10.1038/s41598-023-35866-2)
Supplement: Supplementary file 2 — Supplementary Tables. [file 41598_2023_35866_MOESM2_ESM.docx]

**Development and validation of asthma risk prediction models using co-expression gene modules and machine learning methods**

Eskezeia Y. Dessie^1^, Yadu Gautam^1^, Lili Ding^1^, Mekibib Altaye^1^, Joseph Beyene^2^, Tesfaye B. Mersha^1^

^1^Department of Pediatrics, Cincinnati Children’s Hospital Medical Center, University of Cincinnati College of Medicine, Cincinnati, OH, USA, ^2^Department of Health Research Methods, Evidence, and Impact, McMaster University, Hamilton, Canada

**Table S1**: Summary of genes in clinically significant modules obtained from AECs and NECs datasets

| **AEC data** | | | **NEC data** | | |
| --- | --- | --- | --- | --- | --- |
| Module | # Correlated genes in the module | # DCEGs in the module | Module | # Correlated genes in the module | # DEGs in the module |
| greenyellow | 86 | 59 | blue | 1225 | 425 |
| green | 455 | 112 | brown | 961 | 272 |
| brown | 790 | 68 | black | 237 | 7 |
| purple | 170 | 132 | pink | 211 | 21 |
| magenta | 244 | 144 |  |  |  |
| pink | 283 | 163 |  |  |  |
| yellow | 467 | 176 |  |  |  |
| **Total** | **2495** | **854** |  | **2634** | **725** |

**Table S2**. List of significant pathways for the genes that were correlated with the asthma-associated black, turquoise and yellow co-expression module eigenvectors derived from AECs dataset. The uniquely correlated genes of each module with p-value < 0.01were included as input for pathway analysis

| **Purple module (132 uniquely correlated genes)** | | |
| --- | --- | --- |
| ***Ingenuity Canonical Pathways*** | ***-log(p-value)*** | ***Enriched genes*** |
| **IL-13 Signaling Pathway** | 2.45 | CCL26,CLCA1,MAPK13,POSTN |
| Role of IL-17A in Arthritis | 2.45 | MAP2K6,MAPK13,NOS2 |
| Glutamate Removal from Folates | 2.27 | GGH |
| Histamine Biosynthesis | 2.27 | HDC |
| Adenine and Adenosine Salvage VI | 2.27 | ADK |
| Vitamin-C Transport | 2.17 | GJB2,LRRC8D |
| Role of JAK family kinases in IL-6-type Cytokine Signaling | 2.05 | **BIRC5,MAPK13,TIMP1** |
| ***Pink module (163 uniquely correlated genes)*** | | |
| **Ingenuity Canonical Pathways** | **-log(p-value)** | **Enriched genes** |
| Integrin Signaling | 3.28 | ARF3,ARF6,CAPN1,HRAS,NCK2,PIK3CD,RHOF |
| Mitochondrial Dysfunction | 3.01 | COX7A1,FIS1,GPX4,NDUFB6,NDUFS7,NDUFS8 |
| Ferroptosis Signaling Pathway | 2.74 | ARF3,ARF6,GPX4,H2BC8,HRAS |
| FLT3 Signaling in Hematopoietic Progenitor Cells | 2.67 | BAD,EIF4EBP1,HRAS,PIK3CD |
| **PI3K/AKT Signaling** | 2.67 | BAD,EIF4EBP1,GYS1,HRAS,MAPK8IP1,PIK3CD |
| Insulin Receptor Signaling | 2.62 | BAD,EIF4EBP1,GYS1,HRAS,PIK3CD |
| HER-2 Signaling in Breast Cancer | 2.4 | ARF3,ARF6,BAD,COX7A1,HRAS,PIK3CD |
| Melanoma Signaling | 2.36 | BAD,HRAS,PIK3CD |
| UVB-Induced MAPK Signaling | 2.31 | BAD,EIF4EBP1,PIK3CD |
| **Apoptosis Signaling** | 2.3 | BAD,CAPN1,ENDOG,HRAS |
| Paxillin Signaling | 2.26 | ARF6,HRAS,NCK2,PIK3CD |
| Estrogen Receptor Signaling | 2.23 | BAD,EIF4EBP1,GNB2,HRAS,NDUFB6,NDUFS7,NDUFS8,PIK3CD |
| Oxidative Phosphorylation | 2.2 | COX7A1,NDUFB6,NDUFS7,NDUFS8 |
| Cancer Drug Resistance By Drug Efflux | 2.18 | ABCC2,HRAS,PIK3CD |
| Endometrial Cancer Signaling | 2.14 | BAD,HRAS,PIK3CD |
| Virus Entry via Endocytic Pathways | 2.11 | AP2M1,AP3S2,HRAS,PIK3CD |
| ERB2-ERBB3 Signaling | 2.04 | BAD,HRAS,PIK3CD |
| **Greenyellow module (59 uniquely correlated genes)** | | |
|  | | |
| **Ingenuity Canonical Pathways** | **-log(p-value)** | **Enriched genes** |
| Uracil Degradation II | 2.19 | DPYS |
| Thymine Degradation | 2.19 | DPYS |
| Hepatic Cholestasis | 2.08 | ABCG5,ADCY2,TJP2 |
| **Green (112 uniquely correlated genes)** | | |
| **Ingenuity Canonical Pathways** | **-log(p-value)** | **Enriched genes from input** |
| GDP-mannose Biosynthesis | 3.5 | GMPPB,PMM2 |
| Colanic Acid Building Blocks Biosynthesis | 2.73 | GMPPB,PMM2 |
| Pyrimidine Deoxy ribonucleotides De Novo Biosynthesis I | 2.3 | APOBEC3G,NME4 |
| TCA Cycle II (Eukaryotic) | 2.3 | IDH3G,MDH2 |
| **Brown module (68 uniquely correlated genes)** | | |
| **Ingenuity Canonical Pathways** | **-log(p-value)** | **Enriched genes** |
| Oxytocin In Spinal Neurons Signaling Pathway | 2.44 | ABCC9,NPR3 |
| Tyrosine Biosynthesis IV | 2.26 | PAH |
| Phenylalanine Degradation I (Aerobic) | 2.12 | PAH |

**Table S3**. List of significant pathways for the genes that were correlated with the asthma-associated black, turquoise and yellow co-expression module eigenvectors derived from NECs dataset. The uniquely correlated genes of each module with p-value < 0.01 were included as input for pathway analysis.

| **Brown-module ( 272 uniquely correlated genes)** | | |
| --- | --- | --- |
| **Ingenuity Canonical Pathways** | **-log(p-value)** | **Enriched genes** |
| Pathogen Induced Cytokine Storm Signaling Pathway | 17.5 | CASP1,CCL8,CCR6,CD40LG,CGAS,CIITA,COL6A1,CX3CL1,CXCL13,FASLG,GZMB, |
|  |  | HLA-DOA,HLA-DPA1,HLA-DPB1,IFIH1,IFNG,IL17A,IL21R,IL23R,IRF1,LTA,NLRC3, |
|  |  | NLRC5,PRF1,STAT4,TBX21,TLR10,TLR7,TNF,TNFSF14 |
| Th1 Pathway | 11.6 | CD3D,CD3G,CD40LG,CD80,HLA-DOA,HLA-DPA1,HLA-DPB1,ICOS,IFNG,IRF1,JAK3, |
|  |  | KLRD1,LTA,STAT4,TBX21 |
| Th1 and Th2 Activation Pathway | 11.5 | CD3D,CD3G,CD40LG,CD80,CXCR6,GFI1,HLA-DOA,HLA-DPA1,HLA-DPB1,ICOS,IFNG, |
|  |  | IRF1,JAK3,KLRD1,LTA,STAT4,TBX21 |
| Crosstalk between Dendritic Cells and Natural Killer Cells | 11 | CD226,CD40LG,CD80,FASLG,IFNG,IL15RA,ITGAL,KLRD1,LTA,NCR3,PRF1,TLR7,TNF |
| Multiple Sclerosis Signaling Pathway | 10.7 | CASP1,CD40LG,CTLA4,FASLG,HLA-DOA,HLA-DPA1,HLA-DPB1,IFNG,IL17A,IRF1,LTA, |
|  |  | PARP14,PARP15,SLC8A1,TLR10,TLR7,TNF,TNFSF14 |
| Macrophage Classical Activation Signaling Pathway | 9.83 | ACOD1,CD40LG,CD80,CIITA,FASLG,GBP2,HLA-DOA,HLA-DPA1,HLA-DPB1,IFNG,IL17A |
|  |  | ,IRF1,LTA,PARP14,TNF,TNFSF14 |
| Natural Killer Cell Signaling | 9.53 | CD226,FASLG,FCGR3A/FCGR3B,IFNG,ITGAL,JAK3,KLRB1,KLRC2,KLRD1,LAT,LILRB1, |
|  |  | MRAS,NCR3,SH2D1A,STAT4,ZAP70 |
| Th2 Pathway | 8.7 | CD3D,CD3G,CD80,CXCR6,GFI1,HLA-DOA,HLA-DPA1,HLA-DPB1,ICOS,IFNG,J |
|  |  | AK3,STAT4,TBX21 |
| Phagosome Formation | 7.12 | ACKR4,ADRA2C,AP1S2,CALCR,CCR2,CCR6,CXCR6,FCGR1A,FCGR2B,FCGR3A/FCGR3B, |
|  |  | FZD2,GPR174,ITGAE,ITGAL,LAT,MRAS,MYH7,NMUR1,P2RY11,P2RY12,PLA2G4C,PLD4, |
|  |  | PTGIR,TLR10,TLR7 |
| Antigen Presentation Pathway | 6.86 | CIITA,HLA-DOA,HLA-DPA1,HLA-DPB1,IFNG,NLRC5,PSMB9 |
| Neuroinflammation Signaling Pathway | 6.63 | BIRC3,CASP1,CD80,CX3CL1,FASLG,HLA-DOA,HLA-DPA1,HLA-DPB1,IFNG,JAK3, |
|  |  | NAIP,PLA2G4C,S100B,TLR10,TLR7,TNF |
| Pyroptosis Signaling Pathway | 6.27 | CASP1,GBP1,GBP2,GBP5,GZMA,NAIP,TLR10,TLR7,TNF |
| Role of Pattern Recognition Receptors in Recognition of Bacteria and Viruses | 6.13 | CASP1,CD40LG,CLEC6A,FASLG,IFIH1,IFNG,IL17A,LTA,TLR7,TNF,TNFSF14 |
| TREM1 Signaling | 5.87 | CASP1,CIITA,FCGR2B,NLRC3,NLRC5,TLR10,TLR7,TNF |
| PD-1, PD-L1 cancer immunotherapy pathway | 5.75 | CD80,HLA-DOA,HLA-DPA1,HLA-DPB1,IFNG,JAK3,LAT,TNF,ZAP70 |
| Primary Immunodeficiency Signaling | 5.59 | ADA,CD3D,CD40LG,CIITA,ICOS,JAK3,ZAP70 |
| Airway Pathology in Chronic Obstructive Pulmonary Disease | 5.4 | CD40LG,FASLG,GZMB,IFNG,IL17A,LTA,PRF1,TNF,TNFSF14 |
| Immunogenic Cell Death Signaling Pathway | 5.35 | CASP1,CGAS,GZMA,GZMB,GZMM,IFNG,PRF1,TNF |
| Role Of Osteoclasts In Rheumatoid Arthritis Signaling Pathway | 4.65 | ADAM23,BIRC3,CALCR,COL6A1,FCGR1A,FCGR2B,FCGR3A/FCGR3B, |
|  |  | FOXO6,IFNG,IL17A,MRAS,NAIP,TNF |
| Role Of Osteoblasts In Rheumatoid Arthritis Signaling Pathway | 4.28 | BMP7,CD40LG,FASLG,FZD2,IFNG,IL17A,JAK3,LTA,STAT4,TNF,TNFSF14 |
| CDX Gastrointestinal Cancer Signaling Pathway | 4.28 | BMP7,CD40LG,FASLG,FZD2,IFNG,IL17A,JAK3,LTA,TNF,TNFSF14 |
| Role of PKR in Interferon Induction and Antiviral Response | 4.05 | ATF3,CASP1,FASLG,FCGR1A,IFIH1,IFNG,IRF1,TNF |
| Erythropoietin Signaling Pathway | 4 | BIRC3,CD40LG,FASLG,IFNG,IL17A,LTA,MRAS,TNF,TNFSF14 |
| Tumoricidal Function of Hepatic Natural Killer Cells | 3.99 | FASLG,GZMB,ITGAL,PRF1 |
| T Helper Cell Differentiation | 3.89 | CD3D,CD3G,CD40LG,CD80,HLA-DOA,HLA-DPA1,HLA-DPB1,ICOS,IFNG, |
|  |  | IL17A,IL21R,IL23R,STAT4,TBX21,TNF |
| S100 Family Signaling Pathway | 3.76 | ABCB1,ACKR4,ADRA2C,CACNA1I,CALCR,CCR2,CCR6,CXCR6,FCGR1A, |
|  |  | FCGR2B,FCGR3A/FCGR3B,FZD2,GPR174,NMUR1,P2RY11,P2RY12,PLA2G4C,PTGIR,S100B,TNF |
| Regulation Of The Epithelial Mesenchymal Transition By Growth Factors Pathway | 3.73 | CD40LG,DOCK10,ETS1,FASLG,JAK3,LTA,MRAS,TNF,TNFSF14 |
| IL-10 Signaling | 3.67 | CD80,FCGR2B,HLA-DOA,HLA-DPA1,HLA-DPB1,IFNG,INPP5D,TNF |
| MSP-RON Signaling In Macrophages Pathway | 3.61 | CIITA,HLA-DOA,HLA-DPA1,HLA-DPB1,IFNG,MRAS,TNF |
| T Cell Receptor Signaling | 3.59 | CD3D,CD3G,CD80,CTLA4,FYB1,HLA-DOA,HLA-DPA1,HLA-DPB1,ICOS, |
|  |  | IFNG,ITGAL,LAT,MRAS,PAG1,PTPN22,TNF,ZAP70 |
| Role of Hypercytokinemia/hyperchemokinemia in the Pathogenesis of Influenza | 3.56 | CASP1,IFIT3,IFNG,IL17A,TLR7,TNF |
| TNFR2 Signaling | 3.49 | BIRC3,LTA,NAIP,TNF |
| HMGB1 Signaling | 3.44 | CD40LG,FASLG,IFNG,IL17A,LTA,MRAS,TNF,TNFSF14 |
| Agranulocyte Adhesion and Diapedesis | 3.44 | ACKR4,CCL8,CCR2,CCR6,CX3CL1,CXCL13,CXCR6,MYH7,TNF |
| G-Protein Coupled Receptor Signaling | 3.38 | ACKR4,ADCY7,ADRA2C,CALCR,CCR2,CCR6,CXCR6,FOXO6,FZD2, |
|  |  | GPR174,MEF2C,MRAS,MYLPF,NMUR1,P2RY11,P2RY12,PDE1B,PTGIR |
| Atherosclerosis Signaling | 3.31 | CCR2,CD40LG,IFNG,PLA2G4C,SAA4,TNF,TNFSF14 |
| Death Receptor Signaling | 3.3 | BIRC3,FASLG,NAIP,PARP14,PARP15,TNF |
| IL-12 Signaling and Production in Macrophages | 3.25 | CD40LG,IFNG,IL23R,IRF1,SAA4,STAT4,TNF |
| IL-17 Signaling | 3.11 | CD40LG,FASLG,IFNG,IL17A,LTA,MRAS,TNF,TNFSF14 |
| Granulocyte Adhesion and Diapedesis | 3.08 | ACKR4,CCL8,CCR2,CCR6,CX3CL1,CXCL13,CXCR6,TNF |
| T Cell Exhaustion Signaling Pathway | 3.04 | BATF,CD3D,CD3G,CD80,CTLA4,GZMB,HLA-DOA,HLA-DPA1,HLA-DPB1, |
|  |  | IFNG,JAK3,MRAS,STAT4,TBX21,ZAP70 |
| PTEN Signaling | 2.99 | FASLG,FOXO6,INPP5D,INPP5J,ITGAE,ITGAL,MRAS |
| **PI3K/AKT Signaling** | 2.92 | IL15RA,IL21R,INPP5D,INPP5J,ITGAE,ITGAL,JAK3,MRAS |
| Macrophage Alternative Activation Signaling Pathway | 2.9 | CIITA,CXCL13,FCGR2B,HLA-DOA,HLA-DPA1,HLA-DPB1,JAK3,TNF |
| Wound Healing Signaling Pathway | 2.88 | CD40LG,COL6A1,FASLG,IFNG,IL17A,LTA,MRAS,TNF,TNFSF14 |
| IL-23 Signaling Pathway | 2.88 | IL17A,IL23R,STAT4,TNF |
| Dendritic Cell Maturation | 2.83 | CD1D,CD3D,CD3G,CD40LG,CD80,FCGR1A,FCGR2B,FCGR3A/FCGR3B,HLA-DOA, |
|  |  | HLA-DPA1,HLA-DPB1,IL32,LTA,STAT4,TNF |
| Fc Epsilon RI Signaling | 2.83 | INPP5D,INPP5J,LAT,MRAS,PLA2G4C,TNF |
| Systemic Lupus Erythematosus In B Cell Signaling Pathway | 2.8 | CD40LG,FASLG,FCGR2B,FOXO6,IFIH1,IFIT3,IFNG,IL17A,INPP5D,INPP5J, |
|  |  | LTA,MRAS,PAG1,PIK3AP1,TLR7,TNF,TNFSF14 |
| Differential Regulation of Cytokine Production in Intestinal Epithelial Cells by IL-17A and IL-17F | 2.78 | IFNG,IL17A,TNF |
| CREB Signaling in Neurons | 2.75 | ACKR4,ADCY7,ADRA2C,CACNA1I,CALCR,CCR2,CCR6,CXCR6,FZD2,GPR174, |
|  |  | MRAS,NMUR1,P2RY11,P2RY12,PTGIR |
| CTLA4 Signaling in Cytotoxic T Lymphocytes | 2.74 | AP1S2,CD3D,CD3G,CD80,CTLA4,FYB1,HLA-DOA,HLA-DPA1,HLA-DPB1,IDO1, |
|  |  | ITGAL,LAT,MRAS,PLD4,ZAP70 |
| Role of Cytokines in Mediating Communication between Immune Cells | 2.62 | IFNG,IL17A,IL32,TNF |
| Role of Macrophages, Fibroblasts and Endothelial Cells in Rheumatoid Arthritis | 2.59 | FCGR1A,FCGR3A/FCGR3B,FZD2,IL17A,IL32,LTA,MRAS,TLR10,TLR7,TNF |
| Type I Diabetes Mellitus Signaling | 2.57 | CD3D,CD3G,CD80,FASLG,GZMB,HLA-DOA,HLA-DPA1,HLA-DPB1, |
|  |  | IFNG,IRF1,LTA,PRF1,TNF |
| Cardiomyocyte Differentiation via BMP Receptors | 2.57 | BMP7,MEF2C,MYH7 |
| Role of Osteoblasts, Osteoclasts and Chondrocytes in Rheumatoid Arthritis | 2.57 | BIRC3,BMP7,CALCR,FZD2,IFNG,IL17A,NAIP,TNF |
| Fcγ Receptor-mediated Phagocytosis in Macrophages and Monocytes | 2.52 | FCGR1A,FCGR3A/FCGR3B,FYB1,INPP5D,PLD4 |
| Glucocorticoid Receptor Signaling | 2.49 | CD3D,CD3G,FCGR1A,HLA-DOA,HLA-DPA1,HLA-DPB1,IFNG,IL15RA, |
|  |  | IL21R,JAK3,KRT2,MRAS,PLA2G4C,TNF |
| Retinoic acid Mediated Apoptosis Signaling | 2.46 | IFNG,IRF1,PARP14,PARP15 |
| **Apoptosis Signaling** | 2.34 | BIRC3,FASLG,MRAS,NAIP,TNF |
| Allograft Rejection Signaling | 2.29 | CD3D,CD3G,CD40LG,CD80,FASLG,GZMB,HLA-DOA,HLA-DPA1, |
|  |  | HLA-DPB1,IFNG,PRF1,TNF |
| **Blue-module (425 uniquely correlated genes)** | | |
| **Ingenuity Canonical Pathways** | **P-value** | **Enriched genes** |
| cAMP-mediated signaling | 5.64 | ADCY10,ADCY2,ADORA1,ADRA2A,CALML5,CHRM1,DRD1,  HTR1D,HTR7,MPPED2,PDE5A,PDE9A,PKIB,RAP1GAP,RGS4,S1PR1 |
| G-Protein Coupled Receptor Signaling | 5.14 | ACKR3,ADCY10,ADCY2,ADORA1,ADRA2A,CALML5,CHRM1,DRD1,  FZD10,GNG11,GPR146,GPR85,GPRC5D,GRK5,HCRTR1,HTR1D,HTR7,  KCNH2,MPPED2,PDE5A,PDE9A,PIK3C2G,PTGDR2,RAP1GAP,RGS4,  RRAS,S1PR1,SSTR5,TACR1 |
| S100 Family Signaling Pathway | 4.8 | ACKR3,ADORA1,ADRA2A,BDNF,CALML5,CHRM1,DLC1,DRD1,FZD10,  GPR146,GPR85,GPRC5D,HCRTR1,HNF1A,HTR1D,HTR7,MMP10,MMP28,  NOX4,NTRK2,PGF,PIK3C2G,PLA2G4E,PTGDR2,S1PR1,SERPINF1,SERPINF2,  SSTR5,TACR1,TPM2 |
| CREB Signaling in Neurons | 4.49 | ACKR3,ADCY10,ADCY2,ADORA1,ADRA2A,BMP6,CALML5,CHRM1,DRD1,  FZD10,GNG11,GPR146,GPR85,GPRC5D,HCRTR1,HTR1D,HTR7,NTRK1,NTRK2,  PIK3C2G,PTGDR2,RRAS,S1PR1,SSTR5,TACR1 |
| Serotonin Receptor Signaling | 4.03 | ADCY10,ADCY2,HTR1D,HTR7,SLC18A1,SLC18A2 |
| IL-13 Signaling Pathway | 3.86 | ALOX15B,ANO1,CCL26,CLCA1,DEFB1,IL17RB,PIK3C2G,POSTN,SPDEF |
| Breast Cancer Regulation by Stathmin1 | 3.78 | ACKR3,ADORA1,ADRA2A,BMP6,CHRM1,DRD1,FZD10,GNG11,GPR146,  GPR85,GPRC5D,HCRTR1,HTR1D,HTR7,PGF,PIK3C2G,PPP1R3C,PTGDR2,  RRAS,S1PR1,SPDEF,SSTR5,TACR1 |
| Synaptogenesis Signaling Pathway | 3.57 | ADCY10,ADCY2,BDNF,CALML5,CDH26,EPHA3,EPHA4,NLGN4X,NRXN3,  NTRK2,PIK3C2G,RRAS,STX1B,SYT2,SYT6 |
| Thyroid Cancer Signaling | 3.47 | BDNF,GDNF,HNF1A,NTRK1,NTRK2,PIK3C2G,RRAS |
| Axonal Guidance Signaling | 3.44 | ADAMTS17,ADAMTS9,BDNF,BMP6,CXCL12,DPYSL2,EPHA3,  EPHA4,FZD10,GLI1,GNG11,ITGA2B,MMP10,MMP28,NTRK1,  NTRK2,PGF,PIK3C2G,RRAS,SDC2 |
| STAT3 Pathway | 3.37 | BMP6,CISH,IL17RB,IL1RL1,IL1RL2,IL9R,NTRK1,NTRK2,RRAS |
| Dermatan Sulfate Biosynthesis | 3.33 | CHPF,CHST1,CHST5,DSEL,HS3ST4,SULT1C2 |
| Melatonin Degradation I | 3.25 | CYP2A6 (includes others),CYP2C18,CYP2E1,CYP3A4,  SULT1C2,UGT1A6 |
| Bupropion Degradation | 3.15 | CYP2A6 (includes others),CYP2C18,CYP2E1,CYP3A4 |
| Cardiac Hypertrophy Signaling (Enhanced) | 3.1 | ADCY10,ADCY2,ADRA2A,CALML5,FGF11,FGF13,FZD10,  GNG11,IL17RB,IL1RL1,IL1RL2,IL5,IL9R,ITGA2B,MPPED2,PDE5A,  PDE9A,PIK3C2G,RRAS,TNFSF15 |
| Superpathway of Melatonin Degradation | 3.07 | CYP2A6 (includes others),CYP2C18,CYP2E1,CYP3A4,  SULT1C2,UGT1A6 |
| Dermatan Sulfate Biosynthesis (Late Stages) | 2.97 | CHST1,CHST5,DSEL,HS3ST4,SULT1C2 |
| LPS/IL-1 Mediated Inhibition of RXR Function | 2.94 | ALDH1A3,CHST1,CHST5,CYP2A6 ,CYP2C18,CYP2E1,  CYP3A4,HS3ST4,IL1RL1,IL1RL2,PAPSS2,SULT1C2 |
| Chondroitin Sulfate Biosynthesis (Late Stages) | 2.85 | CHPF,CHST1,CHST5,HS3ST4,SULT1C2 |
| GDNF Family Ligand-Receptor Interactions | 2.78 | DOK1,DOK5,GDNF,GFRA3,PIK3C2G,RRAS |
| Neurotrophin/TRK Signaling | 2.73 | BDNF,NTRK1,NTRK2,PIK3C2G,RRAS,SORCS1 |
| Osteoarthritis Pathway | 2.69 | FZD10,GLI1,HNF1A,IL1RL1,IL1RL2,ITGA2B,ITLN1,  MMP10,PGF,PTHLH,SLC39A8 |
| Ephrin Receptor Signaling | 2.68 | CXCL12,DOK1,EPHA3,EPHA4,GNG11,ITGA2B,PGF,  PIK3C2G,RRAS,SDC2 |
| Dopamine Receptor Signaling | 2.67 | ADCY10,ADCY2,DRD1,PPP1R3C,SLC18A1,SLC18A2 |
| Role Of Osteoblasts In Rheumatoid Arthritis Signaling Pathway | 2.58 | CTSC,CTSG,CXCL12,FZD10,HNF1A,IL5,MMP10,MMP28,  PGF,PIK3C2G,TNFSF15 |
| Nicotine Degradation III | 2.57 | CYP2A6,CYP2C18,CYP2E1,CYP3A4,UGT1A6 |
| Chondroitin Sulfate Biosynthesis | 2.57 | CHPF,CHST1,CHST5,HS3ST4,SULT1C2 |
| PEDF Signaling | 2.56 | BDNF,GDNF,HNF1A,PIK3C2G,RRAS,SERPINF1 |
| CDK5 Signaling | 2.51 | ADCY10,ADCY2,BDNF,DRD1,NTRK2,PPP1R3C,RRAS |
| Wound Healing Signaling Pathway | 2.47 | CMA1,COL13A1,COL5A1,IL1RL1,IL1RL2,IL5,MMP10,PGF,  RRAS,TNFSF15,TPSAB1/TPSB2 |
| Inhibition of Matrix Metalloproteases | 2.41 | MMP10,MMP28,RECK,SDC2 |
| Granulocyte Adhesion and Diapedesis | 2.35 | ACKR3,CCL23,CCL26,CXCL12,IL1RL1,IL1RL2,MMP10,MMP28,SDC2 |
| Nicotine Degradation II | 2.32 | CYP2A6 ,CYP2C18,CYP2E1,CYP3A4,UGT1A6 |
| Acetone Degradation I (to Methylglyoxal) | 2.26 | CYP2A6,CYP2C18,CYP2E1,CYP3A4 |
| Phagosome Formation | 2.2 | ACKR3,ADORA1,ADRA2A,CHRM1,DRD1,FZD10,GPR146,  GPR85,GPRC5D,HCRTR1,HTR1D,HTR7,ITGA2B,MRC2,  PIK3C2G,PLA2G4E,PTGDR2,RRAS,S1PR1,SSTR5,TACR1 |
| Estrogen Biosynthesis | 2.19 | CYP2A6 ,CYP2C18,CYP2E1,CYP3A4 |
| Th2 Pathway | 2.1 | HLA-DQB2,IL17RB,IL1RL1,IL5,PIK3C2G,PTGDR2,S1PR1 |
| GPCR-Mediated Integration of Enteroendocrine Signaling Exemplified by an L Cell | 2.09 | ADCY10,ADCY2,ADCYAP1,CCK,SSTR5 |
| Role Of Chondrocytes In Rheumatoid Arthritis Signaling Pathway | 2.03 | CXCL12,IL17RB,IL1RL1,IL1RL2,MMP10,MMP28,PGF |
| VDR/RXR Activation | 2.02 | COL13A1,CYP24A1,CYP27B1,IL1RL1,KLF4 |
|  |  |  |

**Table S4:** Potential genes identified by different machine learning feature selection methods in the AECs data. RF-Random forest, Lasso-least absolute shrinkage and selection operator, RFE-Recursive Feature Elimination and Boruta-Boruta.

| **Methods** | **# Gene** | **Classifier** | **Sensitivity** | **Specificity** | **MCC** | **F1** |
| --- | --- | --- | --- | --- | --- | --- |
| Borutagenes | 30 | SVM | 0.95 | 0.84 | 0.78 | 0.87 |
| Lassogenes | 30 | SVM | 0.98 | 0.97 | 0.94 | 0.97 |
| RFgenenes | 30 | SVM | 0.95 | 0.73 | 0.67 | 0.81 |
| RFEgenes | 30 | SVM | 0.98 | 0.82 | 0.79 | 0.87 |
| Borutagenes | 30 | RF | 0.98 | 0.84 | 0.80 | 0.88 |
| RFgenenes | 30 | RF | 0.91 | 0.86 | 0.75 | 0.86 |
| Lassogenes | 30 | RF | 0.93 | 0.98 | 0.92 | 0.95 |
| RFEgenes | 30 | RF | 0.93 | 0.87 | 0.80 | 0.88 |

**Table S5:** Potential genes identified by different machine learning feature selection methods in the AECs data. RF-Random forest, Lasso-least absolute shrinkage and selection operator, RFE-Recursive Feature Elimination and Boruta-Boruta

| **Methods** | **# Gene** | **Classifier** | **Sensitivity** | **Specificity** | **MCC** | **F1** |
| --- | --- | --- | --- | --- | --- | --- |
| Borutagenes | 34 | SVM | 0.904 | 0.898 | 0.785 | 0.86 |
| Lassogenes | **34** | **SVM** | **1** | **0.993** | **0.99** | **0.993** |
| RFgenenes | 34 | SVM | 0.911 | 0.898 | 0.79 | 0.864 |
| RFEgenes | 34 | SVM | 0.884 | 0.915 | 0.79 | 0.863 |
| Borutagenes | 34 | RF | 0.87 | 0.926 | 0.793 | 0.864 |
| Lassogenes | 34 | **RF** | **0.99** | **1** | **0.97** | **0.98** |
| RFgenenes | 34 | RF | 0.89 | 0.898 | 0.773 | 0.852 |
| RFEgenes | 34 | RF | 0.884 | 0.905 | 0.777 | 0.854 |

**Table S6**: Validation of the diagnostic performance measures gene signature identified by Lasso method. Diagnostic performance different measures including sensitivity, specificity, MCC and F1 score values in multiple tissue datasets.

| **Model** | **Validation** | **Classifier** | **Sensitivity** | **Specificity** | **MCC** | **F1** |
| --- | --- | --- | --- | --- | --- | --- |
| 30-gene signature based model derived from ACEs data | BECs | RF | 0.821 | 0.633 | 0.427 | 0.64 |
|  | WB | RF | 0.575 | 0.65 | 0.188 | 0.4 |
|  | ASM | RF | 0.765 | 0.824 | 0.56 | 0.825 |
|  | NECs | RF | 0.904 | 0.934 | 0.833 | 0.891 |
|  | BECs | SVM | 0.692 | 0.759 | 0.436 | 0.635 |
|  | WB | SVM | 0.494 | 0.752 | 0.22 | 0.41 |
|  | ASM | SVM | 0.647 | 0.824 | 0.444 | 0.746 |
|  | NECs | **SVM** | 0.956 | 0.864 | 0.79 | 0.864 |
| 34-gene signature based model derived from NCEs data | BECs | RF | 0.872 | 0.582 | 0.431 | 0.642 |
|  | WB | RF | 0.644 | 0.635 | 0.23 | 0.429 |
|  | ASM | RF | 0.824 | 0.824 | 0.625 | 0.862 |
|  | AECs | **RF** | 0.907 | 0.774 | 0.67 | 0.812 |
|  | BECs | SVM | 0.872 | 0.595 | 0.442 | 0.648 |
|  | WB | SVM | 0.736 | 0.576 | 0.255 | 0.444 |
|  | ASM | SVM | 0.824 | 0.824 | 0.625 | 0.862 |
|  | AECs | SVM | 0.884 | 0.774 | 0.647 | 0.8 |

AECs: Airway epithelial cells, ASM: Airway smooth muscle, BECs: Bronchial epithelial cells, NECs: Nasal epithelial cells and WB: Whole blood.

**Table S7**. Comparison DEGs+ WGCNA+ML approach with the standard DEGs+ML approach. Four different machine learning methods were applied to evaluate DEG+ML and DEG+WGCNA+Lasso approaches in asthma prediction. RF-Random forest, Lasso-least absolute shrinkage and selection operator, RFE-Recursive Feature Elimination and Boruta-Boruta

| **Methods** | **Gene Number** | **Sensitivity** | **Specificity** | **MCC** | **F1** |
| --- | --- | --- | --- | --- | --- |
| DEGs+Boruta | 10 | **0.95** | 0.89 | 0.83 | 0.9 |
| DEGs+Lasso | 10 | 0.88 | 0.84 | 0.71 | 0.84 |
| DEGs+RF | 10 | 0.86 | 0.87 | 0.73 | 0.84 |
| DEGs+RFE | 10 | 0.91 | **0.94** | **0.84** | **0.91** |
| DEGs_WGCNA_Boruta | 10 | 0.86 | 0.89 | 0.75 | 0.85 |
| DEGs_WGCNA_Lasso | 10 | **0.95** | **0.95** | **0.90** | **0.94** |
| DEGs_WGCNA_RF | 10 | **0.95** | 0.73 | 0.67 | 0.81 |
| DEGs_WGCNA_RFE | 10 | **0.95** | 0.86 | 0.80 | 0.88 |
| DEGs_Boruta | 20 | **0.98** | 0.86 | 0.82 | 0.89 |
| DEGs_Lasso | 20 | 0.93 | 0.89 | 0.81 | 0.89 |
| DEGs_RF | 20 | 0.91 | 0.79 | 0.67 | 0.82 |
| DEGs_RFE | 20 | 0.95 | **0.92** | **0.87** | **0.92** |
| DEGs_WGCNA_Boruta | 20 | 0.93 | 0.86 | 0.77 | 0.87 |
| **DEGs_WGCNA_Lasso** | 20 | **1** | **0.92** | **0.91** | **0.95** |
| DEGs_WGCNA_RF | 20 | 0.81 | 0.87 | 0.69 | 0.81 |
| DEGs_WGCNA_RFE | 20 | 0.95 | 0.87 | 0.81 | 0.89 |
| DEGs_Boruta | 30 | 0.88 | 0.94 | 0.82 | 0.89 |
| DEGs_Lasso | 30 | **0.95** | **0.97** | **0.92** | **0.95** |
| DEGs_RF | 30 | 0.93 | 0.86 | 0.77 | 0.87 |
| DEGs_RFE | 30 | 0.93 | 0.89 | 0.81 | 0.89 |
| DEGs_WGCNA_Boruta | 30 | **0.98** | 0.84 | 0.80 | 0.88 |
| **DEGs_WGCNA_Lasso** | 30 | 0.93 | **0.98** | **0.92** | **0.95** |
| DEGs_WGCNA_RF | 30 | 0.91 | 0.86 | 0.75 | 0.86 |
| DEGs_WGCNA_RFE | 30 | 0.93 | 0.87 | 0.80 | 0.88 |
